# Cystic fibrosis transmembrane regulator correction attenuates heart failure-induced lung inflammation

**DOI:** 10.3389/fimmu.2022.928300

**Published:** 2022-07-28

**Authors:** Franziska E. Uhl, Lotte Vanherle, Anja Meissner

**Affiliations:** ^1^ Department of Experimental Medical Science, Lund University, Lund, Sweden; ^2^ Wallenberg Centre for Molecular Medicine, Lund University, Lund, Sweden; ^3^ Department of Physiology, Institute of Theoretical Medicine, Medical Faculty, University of Augsburg, Augsburg, Germany

**Keywords:** lung, heart failure, cystic fibrosis transmembrane regulator, inflammation, macrophages

## Abstract

Heart failure (HF) affects 64 million people worldwide. Despite advancements in prevention and therapy, quality of life remains poor for many HF patients due to associated target organ damage. Pulmonary manifestations of HF are well-established. However, difficulties in the treatment of HF patients with chronic lung phenotypes remain as the underlying patho-mechanistic links are still incompletely understood. Here, we aim to investigate the cystic fibrosis transmembrane regulator (CFTR) involvement in lung inflammation during HF, a concept that may provide new mechanism-based therapies for HF patients with pulmonary complications. In a mouse model of HF, pharmacological CFTR corrector therapy (Lumacaftor (Lum)) was applied systemically or lung-specifically for 2 weeks, and the lungs were analyzed using histology, flow cytometry, western blotting, and qPCR. Experimental HF associated with an apparent lung phenotype characterized by vascular inflammation and remodeling, pronounced tissue inflammation as evidenced by infiltration of pro-inflammatory monocytes, and a reduction of pulmonary CFTR+ cells. Moreover, the elevation of a classically-activated phenotype of non-alveolar macrophages coincided with a cell-specific reduction of CFTR expression. Pharmacological correction of CFTR with Lum mitigated the HF-induced downregulation of pulmonary CFTR expression and increased the proportion of CFTR+ cells in the lung. Lum treatment diminished the HF-associated elevation of classically-activated non-alveolar macrophages, while promoting an alternatively-activated macrophage phenotype within the lungs. Collectively, our data suggest that downregulation of CFTR in the HF lung extends to non-alveolar macrophages with consequences for tissue inflammation and vascular structure. Pharmacological CFTR correction possesses the capacity to alleviate HF-associated lung inflammation.

## Introduction

Heart failure (HF) currently affects 64 million people worldwide with increasing prevalence (1). Thus, health care expenditures are substantial; and considering our ageing population, they will continue to rise. HF morbidity and mortality are still high despite remarkable advancements in prevention and therapy (2). Moreover, quality of life remains poor for HF patients (3) as HF causes injury and dysfunction of target organs, including the lung (4–7). Although this affects primary disease management and outcome, the mechanisms underlying target organ injury in HF remain incompletely understood and hence, safe and efficient treatment strategies are limited. Regarding HF-associated lung complications, progress has been made in understanding the pathophysiology of pulmonary oedema, but other pulmonary complications of HF continue to challenge patients and clinicians alike.

Similar to several chronic lung diseases (8), elevated biomarker levels of inflammation are features of chronic HF. An augmentation in pro-inflammatory cytokines, including tumor necrosis factor alpha (TNF-α) (9), has been demonstrated to play a role during HF progression, suggesting an involvement of inflammation during HF-mediated target organ damage (10). We previously showed that therapeutically scavenging TNF-α using Etanercept attenuates target organ dysfunction in a mouse model of HF (7). Therapeutic interventions aimed at limiting TNF-α-mediated inflammation in chronic HF or lung diseases have yielded controversial results (11). Considering this, we invested in understanding the molecular mechanism by which TNF-α signaling promotes target organ function during experimental HF (12). Particularly, we showed that elevated TNF-α levels lead to considerable downregulation of the cystic fibrosis transmembrane regulator (CFTR) in the murine vasculature, heart, brain, and lung tissue (5, 13). The importance of proper CFTR function is appreciated in cystic fibrosis (CF) and chronic obstructive pulmonary disease (COPD). Here, CFTR protein dysfunction is common in the airways of affected patients (14). In contrast to the genetic origin in CF, CFTR dysfunction in COPD is acquired since neutrophil elastase can induce alterations of CFTR expression, which correlate with disease severity (15). Besides epithelial (16) and smooth muscle cells (5, 6), CFTR expression has been documented in several immune cells (17, 18). Peripheral blood monocytes isolated from patients heterozygous for the F508del CFTR mutation showed enhanced interleukin (IL)-8 secretion after activation compared to non-CF controls (19). The latter was corroborated in macrophages isolated from *Cftr* knockout mice (20), suggesting a hyperinflammatory phenotype. Interestingly, pharmacological CFTR inhibition in macrophages increased secretion of pro-inflammatory cytokines (18), suggesting that acquired CFTR dysfunction [e.g., induced by HF, smoking or neutrophil elastase (15, 21, 22)] may contribute to hyperinflammatory immune responses. Since dysregulation of inflammation represents a hallmark of multiorgan manifestations of many diseases, including HF, we tested the hypothesis that murine HF associates with pulmonary CFTR dysfunction and concurrent tissue inflammation, which is correctable by CFTR targeting therapy.

## Material and methods

### Materials

All chemical reagents and solutions were purchased from Fisher Scientific (Gothenburg, Sweden), Saveen & Werner (Limhamn, Sweden) or Sigma-Aldrich (Stockholm, Sweden) unless otherwise stated. Primers for qPCR were purchased from Eurofins (Ebersberg, Germany).

### Animals:

This investigation conforms with the Guide for Care and Use of Laboratory Animals published by the European Union (Directive 2010/63/EU) and the ARRIVE 2.0 guidelines. All animal care and experimental protocols were approved by the institutional animal ethics committee at Lund University (Dnr.: 5.8.18-08003/2017; 5.8.18-04938/2021) and were conducted in accordance with European animal protection laws. Commercially available male wild-type mice (12-14 weeks old; C57BL/6N) were purchased from Taconic (Lyngby, Denmark). All mice were housed under a standard 12h:12h light−dark cycle and had access to standard chow and water *ad libitum*. In the clinic, research into sex differences showed that HF prevalence is about 1.5-2x higher in men above 55 years of age compared to women (23). Moreover, women have a higher probability of survival (24). Females are therefore more protected from HF than males. For this reason, male mice that generally show a stronger phenotype were used in this study.

To ensure blinding, experiments were performed after the animals and samples had received codes that did not reveal the identity of the treatment. HF animals were assigned to vehicle or treatment groups using block randomization. To obey the rules for animal welfare, experimental groups were designed to minimize stress and guarantee maximal information using the lowest group size possible when calculated with a type I error rate of α = 0.05 (5%) and power of 1-β > 0.8 (80%) based on earlier studies (5, 25).

### Myocardial infarction

HF in mice was induced by experimental MI generated by permanent surgical ligation of the left anterior descending (LAD) coronary artery (12). Briefly, mice were anaesthetised with isoflurane (1.5-2% in air), intubated with a 22-gauge angiocatheter, and ventilated with room air at a rate of 120 bpm, 250 µl tidal volume, and 3 cm positive end expiratory pressure. The thorax and pericardium were opened, and the LAD was permanently ligated with 7-0 silk suture (Ågnthos, Sweden). Sham control mice underwent the same procedure without LAD ligation. Mice received pain medication (2 µl/g mouse buprenorphine 0.05 mg/ml *via* intraperitoneal injection) for up to three days post-surgery. This model shows stable cardiac injury 6 weeks after MI (12). CFTR corrector treatment was initiated 10 weeks after MI (**Supplemental Figure 1**). For 2 weeks, mice received daily intraperitoneal (i.p.) injections of Lumacaftor (Lum; 3 mg/kg in DMSO diluted 1:10 with sterile polyethylene glycol (PEG) in deionized (DI) water (50:50)) or were instilled with 50 µl Lum (18 mg/ml in DMSO diluted 1:10 in sterile PBS) 5 times during the treatment period (orotracheal; o.t.). For termination, mice were sedated using inhalation anesthesia (isoflurane 2.5% at 1.5L/min in room air) before cervical dislocation and subsequent trans-cardiac perfusion.

The herein presented investigation comprises data of 3 experimental mouse cohorts with 1) N = 8 for sham and N = 10 for HF, and 2) N = 8 for sham, N = 10 for HF + vehicle, N = 10 for HF + Lum, and 3) N = 6 for HF + Lum i.p., N=8 for HF + Lum o.t. Not all animals were used for histology experiments.

### Cardiac function assessment

Cardiac function was assessed using magnetic resonance (MR) imaging on a 9.4 T MR horizontal MR scanner equipped with Bruker BioSpec AVIII electronics, a quadrature volume resonator coil (112/087) for transmission and a 20 mm linear surface loop coil for reception (Bruker, Ettlingen, Germany), operating with ParaVision 6.0.1. Mice were anaesthetised with isoflurane in room air with 10% oxygen and kept at a respiration of 70-100 bpm and at 36-37°C body temperature (sequence details in supplement). Image-based determination of ejection fraction (EF), stroke volume, cardiac output, end diastolic volume, end systolic volume, and left ventricle mass was performed with Segment (Medviso, Lund, Sweden) (26). Additional details for cardiac function assessment are provided in the Supplementary material online.

### Fluorescence activated cell sorting

After trans-cardiac perfusion, lung-heart blocks were extracted, and a broncho-alveolar lavage was performed by instilling sterile PBS. The left lung was cut into pieces and enzymatically digested in a DNAse-Collagenase XI mix under continuous agitation. After centrifugation, red blood cells were lysed, and the cell pellets were reconstituted in F_c_ block prior to antibody staining (**Supplemental Table 1**). Data acquisition was carried out on a BD LSR II cytometer using FacsDiva software Vision 8.0 (BD Biosciences). Data analysis was performed with FlowJo software (version 10, TreeStar Inc., USA). Cells were plotted on forward (FSC) versus side scatter and single cells were gated on FSC-A versus FSC-H linearity. Pulmonary macrophages were identified as Live, CD45^+^, B220^-^, CD11b^+^, F4/80^+^ cells (gating strategy: **Supplemental Figure 2**). Non-alveolar macrophages were identified as Live, CD45^+^, B220^-^, CD11b^+^, F4/80^+^, SiglecF^-^ cells while alveolar macrophages were identified as Live, CD45^+^, B220^-^, CD11b^+^, F4/80^+^, SiglecF^+^ cells (27–29). For CFTR staining, pulmonary cells were incubated with CFTR antibody and live/dead staining dye without reconstitution in F_c_ block. After washing and centrifugation, cells were resuspended and incubated with a secondary goat anti-mouse AF488 antibody (**Supplemental Table 2**). For cell-specific co-labelling, CFTR antibody was labelled with Alexa Fluor™ 647 NHS ester antibody labelling kit (Invitrogen) and added to the antibody panel for staining and subsequent flow cytometry-based data acquisition.

### Hydroxyproline assay

Hydroxyproline content was measured using the “Hydroxyproline Assay Kit” as per manufacturer’s instructions.

### Cell culture

Murine macrophages (RAW246.7, ATCC TIB-71) were cultivated in high glucose DMEM supplemented with 10% heat inactivated foetal bovine serum and 1% Penicillin/Streptomycin. Cells were activated with 10 ng/ml phorbol 12-myristate 13-acetate (PMA, AdipoGen) for 48 h followed by a 24 h rest period before incubation with 10 µM Lum (Cayman Chemicals) for 24 h. In a second approach, Lum treatment was started at the same time as PMA-induced activation. Cells were harvested after 96 h and subjected to flow cytometry to determine CFTR surface expression.

### Western blot analysis

Lung samples were snap frozen in liquid nitrogen and stored at -80°C until analysis. Samples were homogenised in 1x PBS using an Ultra-Turrax TP18-10 (Janke & Kunkel KG) and proteins lysed in RIPA buffer supplemented with phosphatase and protease inhibitors for 30 min on ice. Afterwards, samples were frozen at -80°C and thawed on ice. Thereafter, protein extracts were centrifuged for 15 min at 20,000 g at 4°C and stored at -20°C. Protein content was measured using the Pierce™ BCA Protein Assay Kit according to manufacturer’s instructions. After SDS-PAGE, proteins were transferred onto PVDF membranes (VWR) using either wet transfer (5 mM Tris, 40 mM glycine, 20% methanol) or semi-dry transfer in TransBlot^®^ Turbo™ (Bio-Rad), blocked with 5% non-fat dry milk powder in PBS-T (1x PBS, 0.05% Tween 20) for 1 h at room temperature and incubated with the respective primary antibody overnight at 4°C. Blots were incubated with secondary, HRP-labelled antibodies for 1-2 h at room temperature and enhanced chemiluminescence was used to visualise proteins using a ChemiDoc™ MP (Bio-Rad). Protein expression was quantified in relation to β-Tubulin expression and normalised to sham animals.

### RNA extraction and quantitative real-time PCR

For total RNA isolation, the right middle lobe was homogenised in 1 ml Trizol (Invitrogen) and isolated according to the manufacturer’s manual. 1 µg of mRNA was reverse transcribed into cDNA using the High-Capacity cDNA Reverse Transcription Kit in an T100TM Thermal Cycler (Bio-Rad). The resulting cDNA was diluted 12.5x and subsequently used for PCR reactions. The PCR protocol consisted of 40 cycles of 30 s denaturation (95°C); 45 s primer annealing (60°C) and 45 s primer extension (72°C) using a CFX384TM Real-Time System with a C1000 TouchTM Thermal Cycler (Bio-Rad). A list of the primers utilised is provided in **Supplemental Table 3**.

### Histology

Lungs were fixed in 4% PFA (Histolab) overnight and transferred into paraffin using a EprediaTM STP 120 Spin Tissue Processor (Fisher Scientific). Afterwards, samples were embedded into paraffin blocks using an EC 350-1 (Especialidades Médicas Myr, S.L.). 4 µm thin sections were cut with a microtome (HM 355S, Thermo Scientific) and fitted onto superfrost glass slides. Paraffin sections were deparaffinised and rehydrated before they were subjected to Haematoxylin & Eosin and Masson-Trichrome staining. Additional details about immunohistochemical staining are provided in the online supplementary material. For quantification, vessel wall thickness of at least 5 vessels per animal from 3-7 animals per group was manually assessed using the “straight line” tool in ImageJ (https://imagej.net/ImageJ) in scale adjusted images. For the qualitative quantification of collagen in Masson-Trichrome stained lung slides, the staining intensity and staining amount in comparable areas of the lungs (mainly around airways and vessels) was graded on a scale from 1-5. At least 5 regions of interest per animal were graded and 8-10 animals per group were evaluated.

For immunofluorescence, paraffin sections were deparaffinized, rehydrated, and subjected to antigen retrieval in 0.1 M sodium citrate buffer (pH 6) for 20 min before blocking with blocking reagent (Roche) and primary antibody incubation in a humidity chamber over night at 4°C. Slides were subsequently washed with PBS, incubated with secondary antibody at RT and mounted with Fluoromount-G with DAPI. Staining was evaluated with a Zeiss Axio Imager and Zen Pro 10 software. For the quantification, MOMA+ and DAPI+ cells in 3 SMA+ vessels from 3 animals were manually counted using ImageJ (https://imagej.net/ImageJ). Positively stained cells for MOMA+ were reported as % of DAPI+ cells per vessel.

### Data and statistical analysis

The data and statistical analysis comply with the recommendations on experimental design and analysis in pharmacology (30). Data were analysed using GraphPad Prism 8 software (San Diego, California). Histology data are expressed as median ± SEM, all other data are expressed as mean ± SEM, where N is the number of animals and n the number of independent measures. Data distribution was determined using Shapiro-Wilk test. For comparisons of 2 independent groups, Student’s t-tests or Mann Whitney tests were used. For comparison of multiple independent groups, one-way analysis of variance (ANOVA) or Kruskal Wallis followed by Tukey or Dunn *post-hoc* testing was used. Treatment effects were determined by performing multiple comparison relative to the HF group with Dunnett’s or Dunn’s *post hoc* testing. Differences were considered significant at p ≤ 0.05. All necessary details on sample size and statistical test results for each figure are provided in **Supplemental Table 4**.

## Results

### The pulmonary phenotype during HF is characterized by vascular remodeling and myeloid cell infiltration

**Figure 1:**
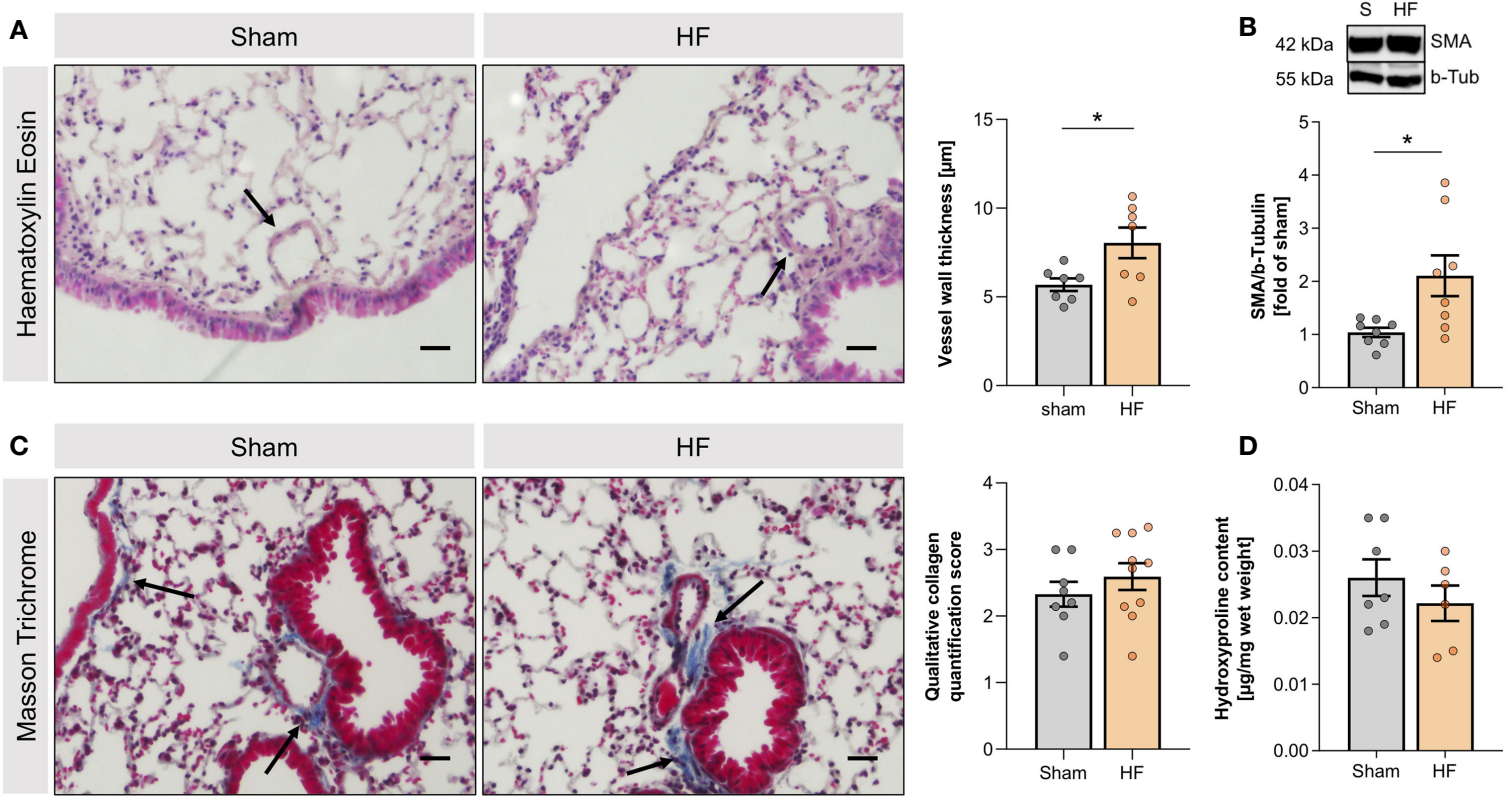
Heart failure-associated structural changes in the lung are confined to blood vessels. **(A)** Representative Haematoxylin and Eosin (H&E) staining of lungs from sham and heart failure (HF) mice (arrows indicate vessel walls; scale bar 20 µm) and quantification of wall thickness of small vessels in H&E-stained lung slices. N=7 per group. **(B)** Representative western blot and quantification of the smooth muscle actin (SMA) protein expression in lung tissue from sham and HF mice. N=8 per group. **(C)** Masson Trichrome (MTC) staining of lungs from sham and HF mice (arrows indicate collagen, stained in blue; scale bar 20 µm) and qualitative quantification of collagen in MTC-stained lung sections. N=8 for Sham, N=10 for HF. **(D)** Quantification of hydroxyproline content of lung tissue from sham and HF mice. N=7 for Sham, N=6 for HF. N denotes the number of mice. Data expressed as mean ± SEM. * denotes p ≤ 0.05 after unpaired t-test.

Twelve weeks after MI, mice presented with cardiac dysfunction evidenced by significantly reduced EF (HF: 43.0% ± 3.3% vs. sham: 64.2% ± 1.8%; **Supplemental Table 5A**) and pulmonary structural alterations confined to the vasculature. HF mice exhibited markedly thicker blood vessel walls (**Figure 1A**) and higher smooth muscle actin (SMA) mRNA (**Supplemental Figure 3**) and protein levels (**Figure 1B**) compared to sham-operated controls. HF lungs did not differ macroscopically nor showed signs of fibrosis demonstrated by the lack of collagen accumulation assessed by Masson trichrome staining (**Figure 1C**) and hydroxyproline quantification (**Figure 1D**).

**Figure 2:**
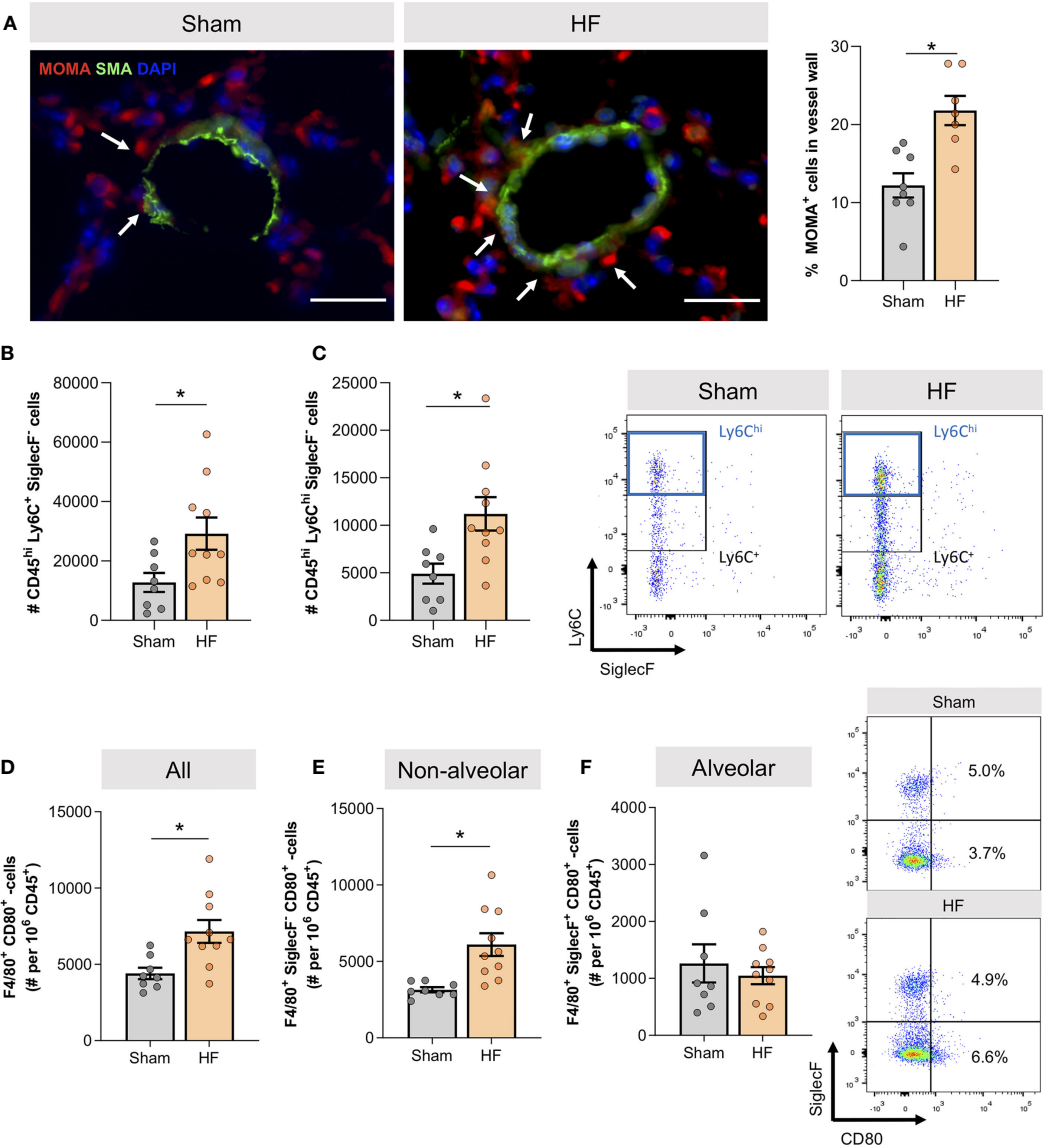
Heart failure associates with lung infiltration of CD80+ pro-inflammatory macrophages. **(A)** Representative images of lung sections from sham and heart failure (HF) mice that were stained for monocyte/macrophages (MOMA) in red, smooth muscle actin (SMA) in green, DAPI stained nuclei in blue. Arrows indicate vessel wall-associated MOMA positivity; scale bar 20 µm. Quantification of the percentage of MOMA positive cells in lung vessel walls. N=3 (n=8) for Sham, N=3 (n=7) for HF. **(B)** Flow cytometry results representing the number of CD45hi Ly6C+ SiglecF- and **(C)** CD45hi Ly6Chi SiglecF- macrophages. N=8 for Sham, N=10 for HF each. Representative dot blots of Ly6C and SiglecF expression of F4/80+ macrophages in the lung of sham and HF mice. **(D)** Flow cytometric assessment of F4/80+ CD80+ classically activated macrophages, **(E)** F4/80+ CD80+ SiglecF- classically-activated non-alveolar macrophages, and **(F)** F4/80+ CD80+ SiglecF+ classically-activated alveolar macrophages in lung tissue of sham and HF mice. Representative dot blots of SiglecF and CD80 expression of F4/80+ macrophages in the lung of sham and HF mice. N=8 for Sham, N=10 for HF each. N denotes the number of mice; n denotes the number of independent measures. Data expressed as mean ± SEM. * denotes p ≤ 0.05 after unpaired t-test.

The apparent vascular remodeling was accompanied by higher monocyte/macrophage association with vascular structures in HF lungs as illustrated by monocyte/macrophage (MOMA) immunostaining in lung slices (**Figure 2A**). Flow cytometric immune cell profiling of the HF lung revealed significantly higher cell numbers of CD45^hi^ Ly6C^+^ SiglecF^-^ cells (**Figure 2B**)and CD45^hi^ Ly6C^hi^ SiglecF^-^ cells in HF compared to sham mice (**Figure 2C** **)**, resembling infiltrating macrophages and pro-inflammatory monocytes. When analyzing the activation profile of F4/80^+^ macrophages, we observed significantly higher cell numbers of classically-activated CD80^+^ macrophages in HF lungs (**Figure 2D**), indicative of a shift to a pro-inflammatory phenotype within the macrophage population. This increase was mainly driven by non-alveolar (SiglecF^-^) macrophages (**Figure 2E**) as no difference was observed in the alveolar (SiglecF^+^) macrophage population (**Figure 2F**).

### Reduced pulmonary CFTR expression is a hallmark of the HF lung

**Figure 3:**
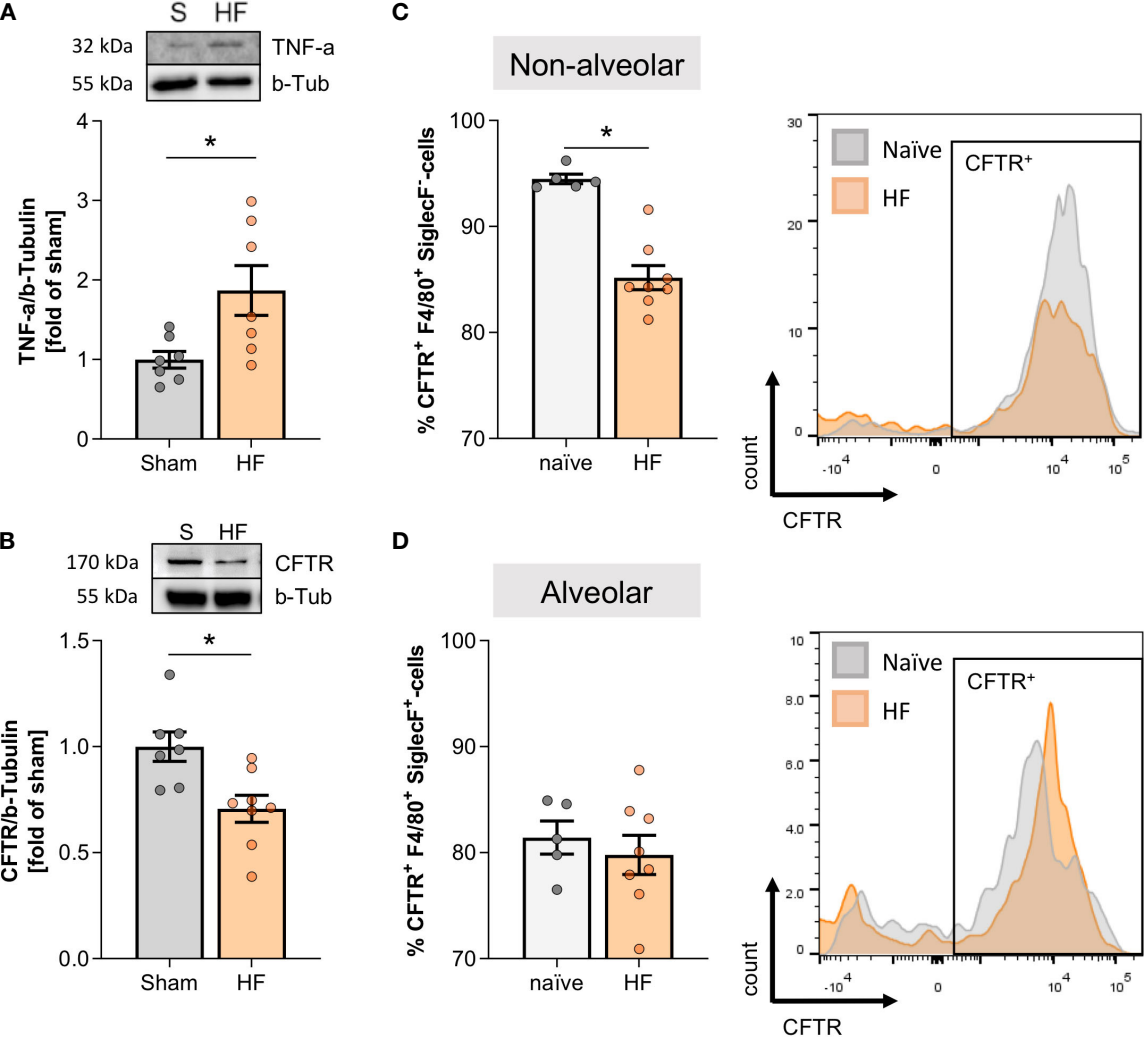
Pulmonary tumour necrosis factor alpha increase is accompanied by decreased cystic fibrosis transmembrane regulator expression in the heart failure lung. **(A)** Representative western blot and quantification of tumour necrosis factor alpha (TNF-α) expression in the lungs of sham and heart failure (HF) mice. N=7 per group. **(B)** Representative western blot and quantification of CFTR protein expression in lungs of sham and HF mice. N=7 for Sham, N=8 for HF. Flow cytometric assessment of proportion of **(C)** CFTR+ F4/80+ SiglecF- non-alveolar macrophages and **(D)** CFTR+ F4/80+ SiglecF+ alveolar macrophages in lung tissue from naïve and HF mice. N=5 for naïve, N=8 for HF each. Representative histograms of CFTR+ SiglecF- and CFTR+ SiglecF+ cells from naïve (grey) and HF (coral) mice. N denotes the number of mice. Data expressed as mean ± SEM. * denotes p ≤ 0.05 after unpaired t-test.

The accumulation of non-alveolar classically-activated macrophages (CD80^+^ SiglecF^-^) associated with markedly higher TNF-α protein levels during HF compared to sham lungs (**Figure 3A**). Since TNF-α potently reduces CFTR surface expression in different cell types (5, 13, 31), we determined CFTR expression with an antibody targeting membrane-associated, mature CFTR protein (32). HF lungs presented with significantly lower expression levels of membrane-bound CFTR assessed by western blotting (**Figure 3B**). Cell-specific CFTR assessment using a flow cytometry approach revealed lower CFTR positivity in SiglecF^-^ non-alveolar macrophages (resembling an infiltrating pro-inflammatory subset) during HF (**Figure 3C**), which coincided with higher CD80 positivity in this subset (see **Figure 2E**). In contrast, no difference in the percentage of CFTR^+^ SiglecF^+^ alveolar macrophage population (resembling resident macrophages) was observed in HF (**Figure 3D**). Different from their non-alveolar counterparts, alveolar macrophages did not upregulate CD80 after MI (see **Figure 2F**).

### Pharmacological CFTR correction mitigates structural changes in the HF lung

**Figure 4:**
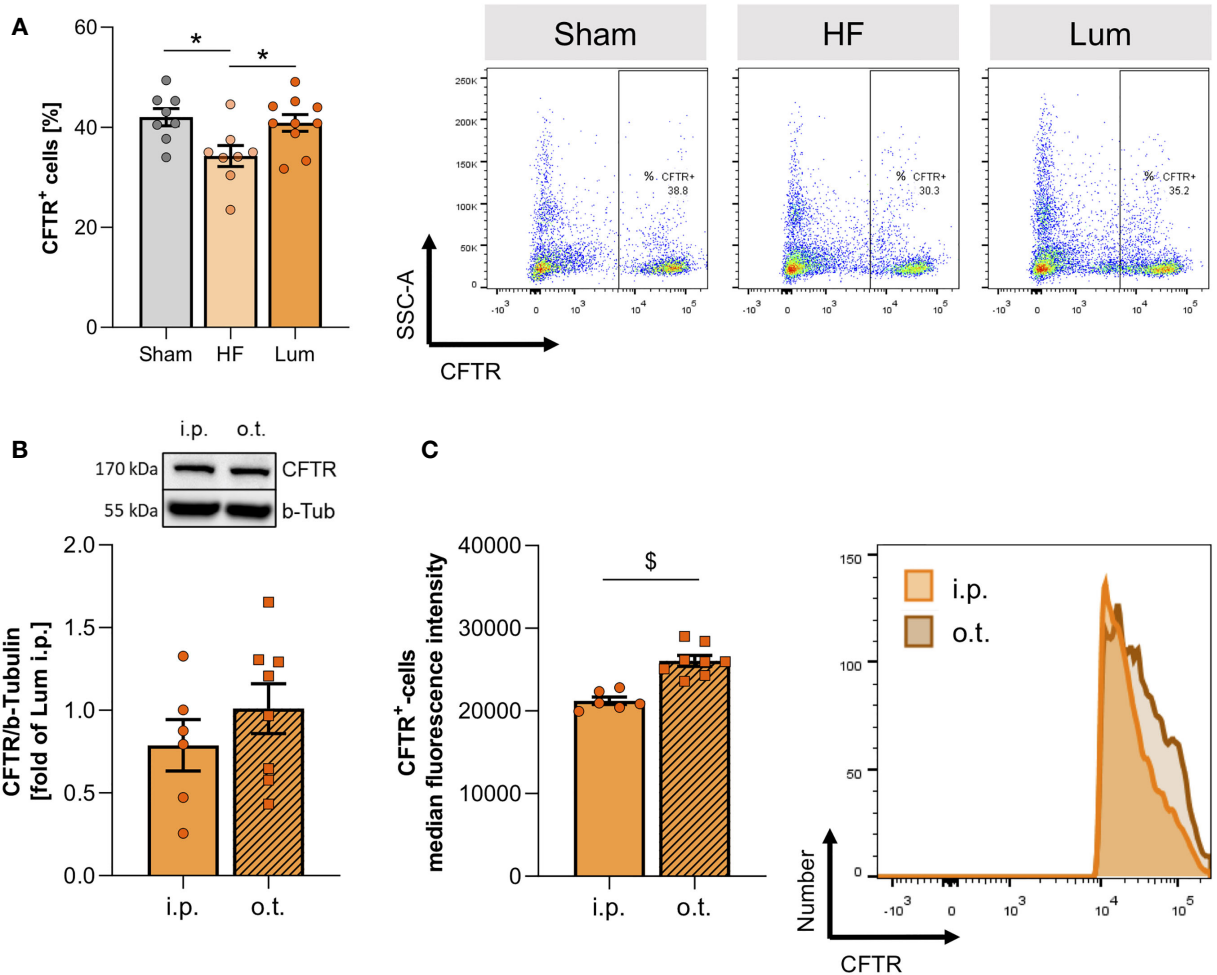
Systemic application of cystic fibrosis transmembrane regulator (CFTR) correctors increases pulmonary CFTR expression. **(A)** Percentage of CFTR+ cells in lungs of sham, heart failure (HF), and Lumacaftor (Lum) treated (intraperitoneally (i.p.)) HF mice and representative dot plots. N=8 for Sham, N=8 for HF + vehicle, N=10 for HF + Lum. **(B)** Representative western blot and quantification of the CFTR expression in the lungs of HF mice treated with Lumacaftor either i.p. or o.t. N=6 for HF + Lum i.p., N=8 for HF + Lum o.t. **(C)** Median fluorescence intensity and representative histograms of CFTR+ cells in the lungs of HF mice treated with Lumacaftor either i.p. (coral) or o.t. (orange). N=6 for HF + Lum i.p., N=8 for HF + Lum o.t. N denotes the number of mice. Data expressed as mean ± SEM. In **(A)**, * denotes p ≤ 0.05 relative to HF after one-way ANOVA with Dunnett’s post-hoc testing; in **(B, C)**, $ denotes p ≤ 0.05 after unpaired t-test.

**Figure 5:**
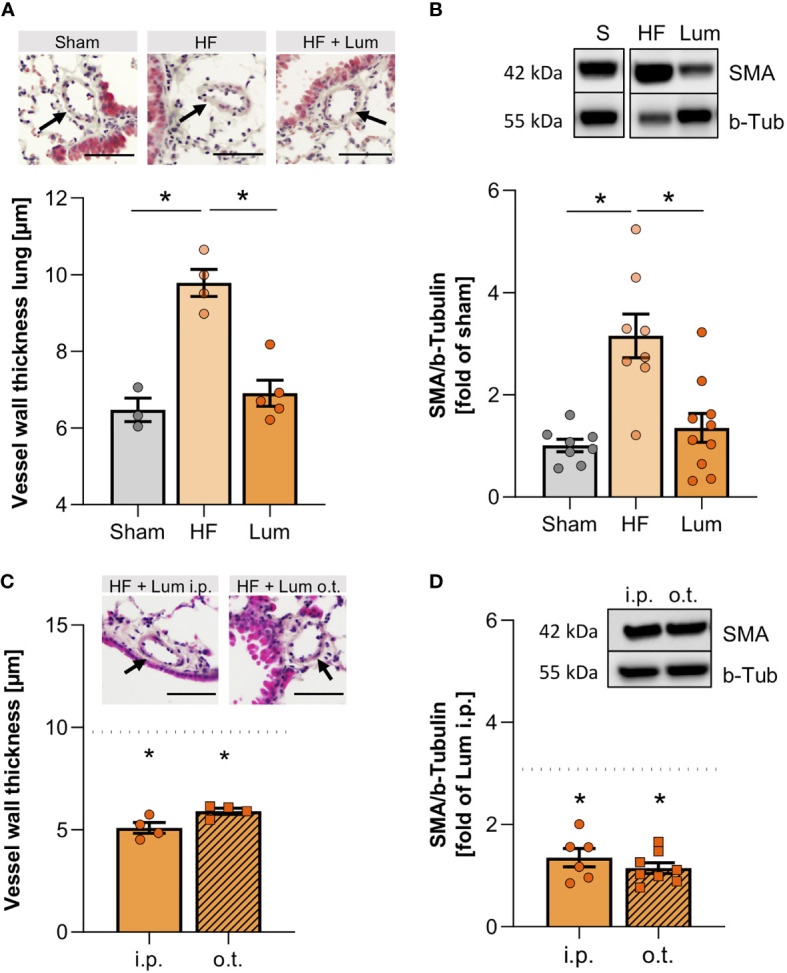
Cystic fibrosis transmembrane regulator correction mitigates heart failure-associated alteration of pulmonary vascular structure. **(A)** Quantification of the vessel wall thickness of smaller vessels in the lungs of sham, heart failure (HF), and Lumacaftor (Lum) treated HF mice. N=3 for Sham, N=4 for HF + vehicle, N=5 for HF + Lum. Insets showing representative images of H&E-stained lung sections; scale bars 50 µm; arrows indicating vessel wall. **(B)** Representative western blot and quantification of the smooth muscle actin (SMA) expression in lung tissue from sham, HF, and Lum treated HF mice. N=8 for Sham, N=8 for HF + vehicle, N=10 for HF + Lum. **(C)** Quantification of the vessel wall thickness of smaller vessels in the lungs of Lum treated (intraperitoneally (i.p.) or orotracheally (o.t.)) HF mice. N=4 for HF + Lum i.p., N=4 for HF + Lum o.t. The dotted line indicates the level of HF mice. Insets showing representative images of H&E-stained lung sections; scale bars 50µm; arrows indicating vessel wall. **(D)** Representative western blot and quantification of SMA expression in lung tissue from Lum treated (i.p. and o.t.) HF mice. N=6 for HF + Lum i.p., N=8 for HF + Lum o.t. The dotted line indicates the level of HF mice. N denotes the number of mice. Data expressed as mean ± SEM. * denotes p ≤ 0.05 relative to HF after one-way ANOVA with Dunnett’s post-hoc testing.

In attempt to improve altered CFTR expression in HF mice, we subjected a group of HF mice to CFTR corrector treatment using Lumacaftor (Lum). Lum acts as a chaperone improving CFTR protein folding and transport to the cell membrane and hence, increases cell surface CFTR protein expression (33, 34). Systemic (i.p.) Lum administration 10 weeks post-MI did not affect heart function (**Supplemental Table 5B**), while significantly increasing the proportion of CFTR^+^ cells in the HF lung (**Figure 4A**). Similarly, western blot evaluation confirmed that the membrane-specific CFTR protein expression was not different from sham levels after two weeks of CFTR corrector treatment (**Supplemental Figure 4**). Lung-specific, orotracheal (o.t.) Lum instillation did not result in higher CFTR protein expression compared to systemic i.p. administration (**Figure 4B**). However, o.t. Lum administration resulted in significantly higher CFTR expression on the cell surface of CFTR^+^ lung cells as evidenced by increased median fluorescence intensity (MFI) in the o.t.-treated lungs compared to lungs from i.p.-treated HF mice (**Figure 4C**). CFTR correction attenuated alteration of the pulmonary vascular structure in the HF lung. Lum application mitigated the HF-associated thickening of pulmonary blood vessel walls (**Figure 5A** and **Supplemental Figure 5**) and led to significantly lower SMA protein levels (**Figure 5B**). This treatment effect was independent of application route supported by similar vessel wall thickness (**Figure 5C**) and SMA protein expression (**Figure 5D**) after both i.p. and o.t. Lum treatment.

### Pharmacological CFTR correction promotes an anti-inflammatory phenotype of macrophages in the HF lung

**Figure 6:**
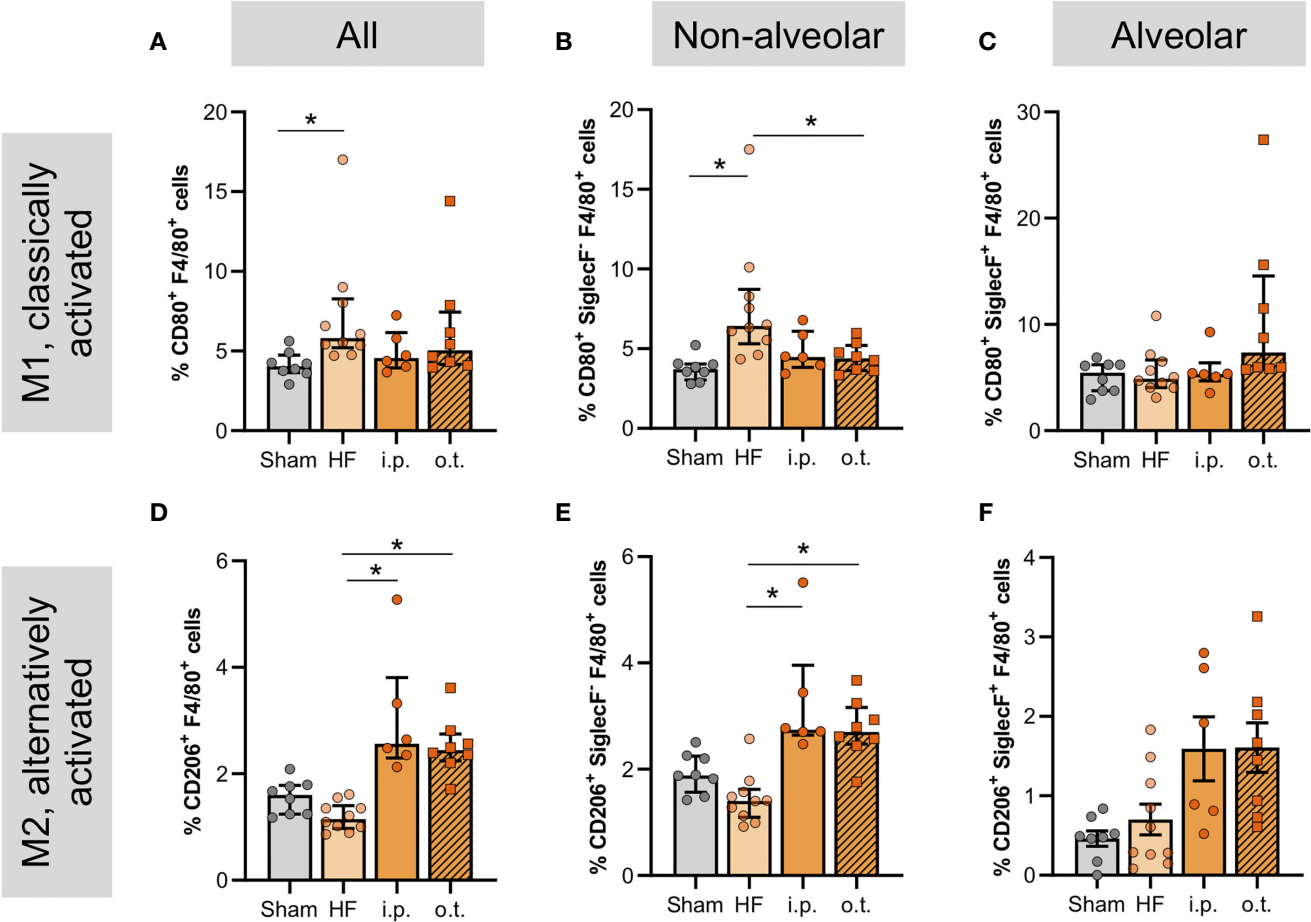
Cystic fibrosis transmembrane regulator correction normalizes levels of non-alveolar macrophages and increases CD206+ alveolar macrophages. Proportion of pulmonary F4/80+-macrophages in sham, heart failure (HF), and Lumacaftor (Lum) treated ((intraperitoneally (i.p.) or orotracheally (o.t.)) HF mice positive for **(A)** CD80 and **(D)** CD206+. N=8 for Sham, N=10 for HF + vehicle, N=6 for HF + Lum i.p., N=8 for HF + Lum o.t. each. Percentage of pulmonary non-alveolar F4/80+ and SiglecF- macrophages in sham, HF, and Lum treated (i.p. and o.t.) HF mice positive for **(B)** CD80+ and **(E)** CD206+. N=8 for Sham, N=10 for HF + vehicle, N=6 for HF + Lum i.p., N=8 for HF + Lum o.t. each. Percentage of pulmonary alveolar F4/80+ and SiglecF+ macrophages in sham, HF, and Lum treated (i.p. and o.t.) HF mice positive for **(C)** CD80+ and **(F)** CD206+. N=8 for Sham, N=10 for HF + vehicle, N=6 for HF + Lum i.p., N=8 for HF + Lum o.t. each. N denotes the number of mice. In **(A, B, D, F)**, data expressed as mean ± SEM; * denotes p ≤ 0.05 relative to HF for multiple comparisons with Dunnett’s post-hoc testing. In **(C, E)**, data expressed as median ± IQR; * denotes p ≤ 0.05 relative to HF for multiple comparisons with Dunn’s post-hoc testing.

Considering the high CFTR positivity of peripheral and pulmonary monocytes and macrophages (**Supplemental Figures 6A,B**), we explored the effects of pharmacological CFTR correction on macrophages in the lung. Both systemic and lung-specific Lum administration significantly increased the overall number of pulmonary macrophages (**Supplemental Figure 7**) with larger effects after o.t. application. The treatment-associated increase of overall pulmonary macrophage counts was mainly mediated by increases of non-alveolar macrophages (**Supplemental Figure 7B**). In contrast to systemic administration, o.t.-administered Lum markedly augmented the number of alveolar macrophages (**Supplemental Figure 7C**). To understand whether this increase in macrophages was beneficial or rather detrimental, we explored macrophage activation profiles by determining the proportion of classically- (CD80^+^) and alternatively- (CD206^+^) activated cells within the pulmonary macrophage population. The HF-associated augmentation of classically-activated macrophages was not significantly alleviated by therapeutic Lum administration when assessing all CD80+ macrophages in the lung (**Figure 6A**), however, therapeutic CFTR correction significantly attenuated the HF-associated increase of non-alveolar CD80^+^ macrophages (**Figure 6B**). Interestingly, o.t.-treated HF lungs presented with higher proportions of CD80^+^ alveolar macrophages but differences did not reach statistical significance (**Figure 6C**). In contrast to CD80^+^ macrophages, Lum induced higher proportions of alternatively-activated (CD206+) macrophages overall (**Figure 6D**) as well as non-alveolar (**Figure 6E**) but not alveolar macrophages (**Figure 6F**) irrespective of treatment route (**Figure 6**, **Supplemental Figure 8** and **Supplemental Table 5**). This is corroborated by increased pulmonary IL-10 mRNA expression after systemic Lum administration (**Supplemental Figure 9**). *In vitro*, murine macrophages (RAW246.7 cells) presented with reduced CFTR positivity after PMA-induced activation, which was attenuated by CFTR correction with Lum (**Supplemental Figure 10**), suggesting an interplay between CFTR surface expression and macrophage activation.

## Discussion

This study describes an apparent lung phenotype during experimental HF characterized by vascular remodeling and pronounced tissue inflammation. Specifically, our data suggest that the elevation of classically-activated non-alveolar macrophages coincides with a cell-specific reduction of pulmonary CFTR expression. In accordance, we show that pharmacological increase of CFTR expression, which increases the proportion of CFTR^+^ cells in the lung, diminishes the HF-associated elevation of classically-activated non-alveolar macrophages, induces the increase of an alternative macrophage polarization, and normalizes vessel wall thickness within the lungs of HF mice. Taken together, our data suggest pharmacological increase of CFTR protein expression to have beneficial effects on the macrophage profile in the HF lung with favorable implications for pulmonary vascular structure. We therefore propose therapeutic CFTR correction as promising approach to alleviate HF-induced inflammation in the lung.

The manifestation of HF in the lung is well-established. However, difficulties in the treatment of HF patients with chronic lung phenotypes remain, as standard therapies are often complicated by contraindications. Here, we verify a HF-mediated CFTR downregulation in the lung (5, 13), a concept that may provide new mechanism-based treatment options for HF patients with pulmonary complications. Given the increasing evidence for an acquired CFTR dysfunction not only during HF but also in classic chronic lung diseases such as COPD and asthma (35), the indication that CFTR modulators may be useful therapeutics in the treatment of acquired CFTR abnormalities is certainly of interest to the field. First trials verified efficacy of the CFTR potentiator ivacaftor in COPD patients with chronic bronchitis (36). Here, we describe beneficial effects of Lum-mediated CFTR protein expression increases on lung inflammation and associated structural alterations during experimental HF. Specifically, Lum therapy attenuated the HF-associated increase in small vessel wall thickness, indicating beneficial effects on pulmonary arteriopathy, which often accompanies HF in patients with chronic left ventricular dysfunction (37), generally associating with increased risk of pulmonary complications and hence, overall poor disease outcome. Despite thickened vessel walls in the HF lung, we did not observe higher collagen accumulation within HF lungs or around the pulmonary vasculature. In our experiments, we aim at obtaining physiological values for animal ventilation during surgery to avoid ventilator-induced lung injury (38), which cannot be excluded from other studies that reported additional structural alterations and higher collagen content in HF lungs in mice with comparable EF (39, 40).

Inflammation is a key player in both chronic heart and lung diseases and critically contributes to vasculopathies. Here, we find increased numbers of pro-inflammatory monocytes/macrophages infiltrating the HF lung and an accumulation of monocytes/macrophages around the pulmonary vasculature, suggesting inflammation-associated vascular remodeling. Monocytes/macrophages have been shown to be among the primary effectors of inflammation in pulmonary lesions, and lung interstitial macrophages play a major role in lung inflammation and dysfunction in several diseases. Monocytes expressing certain chemokine receptors have been shown to differentiate into interstitial perivascular macrophages, which secrete pro-inflammatory cytokines and contribute to vascular remodeling (41). Whether changes in CFTR surface expression on circulating monocytes/macrophages mediates similar effects is an interesting question, especially considering their (1) relatively high CFTR positivity compared to other immune cells (2), reported increased secretion of pro-inflammatory cytokines after pharmacological CFTR inhibition in macrophages (18), the herein observed (3) activation-induced CFTR surface reduction on macrophages and (4) reduction of CFTR^+^ SiglecF^-^ non-alveolar macrophages. Further cell type-specific investigations to characterize the CFTR expression in different cell types after MI could give insight into which cells are mainly affected and benefit from CFTR corrector treatment.

HF leads to systemic TNF-α elevation in mice and men (5, 25, 42), which negatively affects target organs, including the lung (42). We previously showed that TNF-α sequestration with Etanercept attenuated the HF-associated reduction of pulmonary CFTR protein expression (5). TNF-α was shown to mediate reduction of CFTR expression on the surface of different cell types (5, 31), suggesting that the herein detected HF-associated augmentation of pulmonary TNF-α might be directly linked to the observed overall CFTR downregulation in the HF lung. TNF-α, amongst other pro-inflammatory cytokines, induces M1-like macrophage phenotypes (43) and is secreted by classically-polarized CD80^+^ macrophages (44), which accumulate in the HF lung in our model. TNF-α sequestration using Etanercept was shown to reduce M1-type markers supported by decreases of CD40 and CD80 surface markers and increased expression of M2-type markers in human monocyte-derived macrophages (45). Here, we find similar-to-sham levels for CD80^+^ non-alveolar macrophages in the HF lung after Lum therapy, suggesting an intimate link between CFTR signaling and inflammation in the HF lung. Although direct Lum application to the lung resulted in higher CFTR expression on pulmonary CFTR^+^ cells, supporting higher corrector efficacy, higher CD80^+^ alveolar macrophage numbers that were observed with this treatment regimen may limit long-term benefits of lung-specific Lum application. Lum-induced increases of IL-10 in combination with the elevation of CD206^+^ cells in our model are suggestive of an involvement of CFTR in macrophage phenotype switching that promote a more restorative environment (44). An alternative activation of human monocytes from CF patients after CFTR corrector therapy as evidenced by increased IL-10 secretion (46) corroborate our findings. Since CFTR alterations in pulmonary macrophages and monocyte-derived macrophages present with an exaggerated cytokine response to bacterial lipopolysaccharide (20) altered bactericidal activity (47), and adhesion (48), a direct role of CFTR in lung inflammation during HF is likely.

To not only test the effect of increased CFTR membrane expression but also that of increased CFTR channel function, clinically available combination treatments like Trikafta/Kaftrio or Orkambi (i.e., potent CFTR expression correctors and potentiator combinations) should be considered in future studies. We further acknowledge that CFTR therapeutics for the clinic are generally produced for oral administration. The development of CFTR correctors/potentiators for inhalation therapy is rather unlikely. In our experimental approach, we used the oro-tracheal treatment route to clearly distinguish lung-specific from systemic effects of Lum treatment. Our results that reveal overall similar Lum responses after systemic application *via* i.p. injections and oro-tracheal application suggest that systemic applications in the clinic (e.g., through oral treatment routes) may be similarly beneficial in treating lung inflammation during HF.

Taken together, HF presents with an apparent lung phenotype characterized by inflammation and thickened walls of small vessels within the lung and an elevation of classically-activated non-alveolar macrophages that coincides with lower CFTR positivity in this immune cell subset. Pharmacological increase of CFTR expression with Lum lowers HF-associated pro-inflammatory macrophage numbers, while promoting an alternatively-activated phenotype and an attenuation of vascular structural alterations within the HF lung. Collectively, these data suggest pharmacological CFTR correction as promising approach to mitigate HF-induced pulmonary inflammation and associated structural alterations.

## Data availability statement

The original contributions presented in the study are included in the article/supplementary material. Further inquiries can be directed to the corresponding author.

## Ethics statement

This investigation conforms with the Guide for Care and Use of Laboratory Animals published by the European Union (Directive 2010/63/EU) and the ARRIVE 2.0 guidelines. All animal care and experimental protocols were approved by the institutional animal ethics committee at Lund University (Dnr.: 186 5.8.18-08003/2017; 5.8.18-04938/2021) and were conducted in accordance with European and Swedish animal protection laws.

## Author contributions

Conceptualization, AM; methodology, FU and AM; validation, FU and AM; formal analysis, FU and LV; resources, AM; data curation, FU and LV; writing - original draft preparation, FU and AM; writing - review and editing, FU, LV and AM.; visualization, FU, LV and AM; supervision, AM; project administration, AM; funding acquisition, FU and AM. All authors have read and agreed to the published version of the manuscript.

## Funding

This work was supported by the following funding sources: Knut and Alice Wallenberg foundation [F 2015/2112 to AM]; Swedish Research Council (VR) [2017-01243 to AM]; German Research Foundation (DFG) [ME 4667/2-1 to AM]; Åke Wibergs Stiftelse [M19-0380 to AM]; Albert Påhlssons Stiftelse [120482 to AM]; Inger Bendix Stiftelse [2019-10 to AM]; Stohnes Stiftelse [AM]; Crafoord Foundation [20190782 to FU]; Royal Physiographic Society Lund [39716 and 40682 to FU]; STINT [MG19-8469 to AM]; and Lund University [to AM].

## Acknowledgments

The authors thank the Knut and Alice Wallenberg foundation for generous support and the Lund University BioImaging Center (LBIC), Lund University is gratefully acknowledged for providing experimental resources. We further like to thank René In ‘t Zandt and Michael Gottschalk from LBIC for help with the cardiac MRI analyses, Dr. Steffen-Sebastian Bolz and Dr. Darcy Lidington (both Department of Physiology, University of Toronto, Toronto, Canada) for providing lung tissue, Dr. Nicholas Don-Doncow (Department of Experimental Medical Science, Lund University, Lund, Sweden) for discussing FACS panel design, Dr. Gunilla Westergren-Thorsson (Department of Experimental Medical Science, Lund University, Lund, Sweden) for access to the microtome, Dr. Jonas Erjefält (Department of Experimental Medical Science, Lund University Sweden) for access to the paraffin embedding machine, Dr. Darcy Wagner (Department of Experimental Medical Science, Lund University, Lund Sweden) for access to the tissue processor, Dr. Ulrica Englund Johansson (Department of Clinical Sciences Section IV, Lund University, Lund Sweden) for access to the Zeiss Axio Imager, and Dr. Björn Olde (Department of Clinical Sciences Section II, Lund University Sweden) for the RAW246.7 cells.

## Conflict of interest

The authors declare that the research was conducted in the absence of any commercial or financial relationships that could be construed as a potential conflict of interest.

## Publisher’s note

All claims expressed in this article are solely those of the authors and do not necessarily represent those of their affiliated organizations, or those of the publisher, the editors and the reviewers. Any product that may be evaluated in this article, or claim that may be made by its manufacturer, is not guaranteed or endorsed by the publisher.
