# Supplementary material for: Cystic fibrosis transmembrane regulator correction attenuates heart failure-induced lung inflammation
Source: Front Immunol. 2022 Jul 28;13:928300. doi: 10.3389/fimmu.2022.928300 (PMC9365932; doi:10.3389/fimmu.2022.928300)

Supplement

Cystic Fibrosis Transmembrane Regulator Correction Attenuates Heart Failure-Induced Lung Inflammation

Franziska E. Uhl[1,2], Lotte Vanherle[1,2], & Anja Meissner[1,2,3]

[1] Department of Experimental Medical Science, Lund University, Lund, Sweden.
[2] Wallenberg Centre for Molecular Medicine, Lund University, Lund, Sweden.
[3] Department of Physiology, Institute of Theoretical Medicine, Medical Faculty, University of Augsburg, Augsburg, Germany.

Material and Methods:

***Histology*:** Samples for staining with Haematoxylin & Eosin and Masson-Trichrome were fixed in 4% PFA (Histolab) overnight and transferred into paraffin using a Epredia™ STP 120 Spin Tissue Processor (Fisher Scientific). Afterwards, samples were embedded into paraffin blocks using an EC 350-1 (Especialidades Médicas Myr, S.L.). 4 µm thin sections were cut with a microtome (HM 355S, Thermo Scientific) and fitted onto superfrost glass slides. Paraffin sections were deparaffinised and rehydrated before incubation in Meyers Haematoxylin (Histolab) for 10 min, HCl-ethanol (0.3% in 70% ethanol) for 1 min, tap water for 5 min, and Eosin (0.5 g in 70% ethanol) for 1 min. After dehydration, glass slides were covered with coverslips using Rotimount (Roth) and imaged with an Olympus BX60 using the CellSens Dimension 1.5 Software (Olympus). For the determination of the vessel wall thickness at least 3 vessels per animal were measured. Mean value of the thickness at 5 different locations per vessel were used. For each condition at least 4 animals were evaluated in a blinded fashion. For Masson Trichrome staining, a commercially available Kit was used, and the instructions of the manufacturer followed. In addition, slides were fixed in Bouin’s solution (75% saturated Picric acid, 10% Formaldehyde, 5% glacial acetic acid) for 30 min at 56°C after deparaffinization. In short, slides were stained with Weigert’s iron haematoxylin working solution for 20 min, Scarlet Biebrich/acid fuchsin solution for 5 min, phosphotungstic/phosphomoloybdic acid working solution for 15 min, aniline blue solution for 15 min, 1% acetic acid for 2 min and quickly dehydrated and cover slipped. For the qualitative quantification, the staining intensity and staining amount in comparable areas of the lungs (mainly around airways and vessels) was graded on scale from 1-5. At least 5 regions of interest per animal were graded and at least 10 animals per group were evaluated. To minimise staining variations, samples were stained and analysed in batch. Each glass slide contained slices from one animal of each group (sham, HF, HF+Lum).

Cardiac function assessment: Flow compensated FLASH with electrocardiogram (ECG) and respiration triggering (Stony Brook, USA) with a resolution of 0.13x0.13x1mm^3 was used for all MR scans. Positioning of the cardiac images was achieved by three orientational scans: (1)

three axial slices (TR = 50 ms, TE = 2.5 ms), (2) and (3) each with one slice (TR = 6 ms, TE = 2.1 ms, 24 timeframes) orthogonal to each other with slices positioned through the left and right ventricle and through the outflow tract of the left ventricle and the apex, respectively. Short axis view images of 9-10 slices (depending on heart size) were acquired with 24 timeframes in each (TR = 6 ms, TE = 2.1 ms).

SUPPLEMENTAL TABLES

Supplemental Table 1: Primary antibodies used for FACS, immunofluorescence, and Western Blotting. *AF – Alexa Fluor, CD – cluster of differentiation, CFTR – cystic fibrosis transmembrane regulator, FACS- Fluorescence-activated cell sorting, IF – immunofluorescence, MOMA – macrophage/monocyte, SMA – smooth muscle actin, TNF-α – tumour necrosis factor alpha, WB – Western blot.*

Antibody	Company	Ordernr.	Concentration and application	Size [kDa]
β-Tubulin	Sigma	T4026	1:5,000 WB	55
B220 APC Cy7	BD	561102	1:200 FACS	-
B220 AF488	R&D	FAB1217G	1:200 FACS	-
B220 PerCp eF710	Fisher Scientific	46-0452-82	1:200 FACS	-
CD11b PE-Dazzle	BioLegend	101256	1:200 FACS	-
CD206 PE	BioLegend	141705	1:200 FACS	-
CD3 eFluor 450	Fisher Scientific	48-0032-82	1:200 FACS	-
CD31 PE-Cy7	Fisher Scientific	15300920	1:200 FACS	-
CD45 AF700	R&D	FAB114N	1:200 FACS	-
CD45 AF488	R&D	GAB114G	1:200 FACS	-
CD45 PE	Nordic Biosite	109807	1:200 FACS	-
CD80 BV650	BioLegend	104731	1:200 FACS	-
CFTR	Fisher Scientific	MA1-935	1:400 IF and FACS; 1:1,000 WB	170
CFTR AF647 labelled	Fisher Scientific	MA1-935	1:400 FACS	-
epCAM PE	Fisher Scientific	15228839	1:200 FACS	-
F4/80 APC	eBiosciences	17-4801-82	1:200 FACS	-
F4/80 PE	Fisher Scientific	15278779	1:200 FACS	-
Live/Dead Aqua BV510	Fisher Scientific	L34966	1:500 FACS	-
Ly6C PE-Cy7	Fisher Scientific	25-5932-82	1:200 FACS	-
Ly6G APC	Fisher Scientific	17-9668-82	1:200 FACS	-
Ly6G FITC	Fisher Scientific	11-9668-82	1:200 FACS	-
MOMA	GeneTex	GTX39773	1:200 IF	-
SiglecF BV421	BioLegend	155509	1:200 FACS	-
SMA	Sigma	A5228	1:500 IF; 1:5,000 WB	42
TNF-α	Abcam	Ab6671	1:1,000 WB	32

Supplemental Table 2: Secondary antibodies used for FACS, immunofluorescence and Western Blotting. *HRP – horseradish peroxidase, IF – immunofluorescence, WB – western blot.*

Antibody	Company	Ordernr.	Concentration and application
Alexa Fluor 488 goat anti-mouse	Invitrogen	A-11029	1:500 IF, 1:400 FACS
Alexa Fluor 594 goat anti-mouse	Invitrogen	A-11032	1:500 IF
Alexa Fluor 594 goat anti-rat	BioLegend	405422	1:500 IF
HRP-labelled goat anti-mouse	Cell signalling	7076S	1:10,000 WB
HRP-labelled goat anti-rabbit	Cell signalling	7074S	1:10,000 WB

Supplemental Table 3: Primers used for qRT-PCR. *Acta – actin alpha, IL – interleukin*

Gene	Sequence (5' to 3')	Annealing temp. [°C]
Acta2	Fw: GCTGGTGATGATGCTCCCA	58.8
	Rv: GCCCATTCCAACCATTACTCC	59.8
IL-10	Fw: ATGGTGTCCTTTCAATTGCTCT	55.9
	Rv: AGGATCTCCCTGGTTTCTCTTC	58.4
L14	Fw: GGCTTTAGTGGATGGACCCT	59.4
	Rv: ATTGATATCCGCCTTCTCCC	57.3

Supplemental Table 4: Article Figure Data and Statistics. Non-highlighted data sets are means ± SEM; blue-highlighted data sets were subjected to non-parametric statistical analyses and are medians ± interquartile range. Column *"n"* refers to the number of measures; column *"N"* refers to the number of mice. Significant p values are highlighted in red. *CD – cluster of differentiation, CFTR – cystic fibrosis transmembrane regulator, HF – heart failure, IL – interleukin, i.p. – intraperitoneal, Ly6C – lymphocyte antigen 6C, MFI – median fluorescence intensity, MOMA – monocyte-macrophage, o.t. – orotracheal, QCQS – qualitative collagen quantification score, SMA – smooth muscle actin, TNFa – tumour necrosis factor alpha.*

Figure	Parameter	Control	n	N	HF	n	N	Treatment (i.p.)	n	N	Treatment (o.t.)	n	N	Comparison Test	Statistical Outcome
1A	Vessel wall thickness	5,686 ± 0,3545	7	7	8,04 ± 0,865	7	7							unpaired t-test	t(12) = 2.519, **p = 0.0270**
1B	SMA (Protein)	1,039 ± 0,087	8	8	2,106 ± 0,385	8	8							unpaired t-test	t(14) = 2.705, **p = 0.0171**
1C	Collagen QCQS	2,327 ± 0,187	8	8	2,592 ± 0,202	10	10							unpaired t-test	t(16) = 0.9438, p = 0.3593
1D	Hydroxyproline	0,026 ± 0,003	7	7	0,022 ± 0,003	6	6							unpaired t-test	t(11) = 0.9956, p = 0.3408
S3	Acta2 (mRNA)	1,000 ± 0,099	8	8	1,964 ± 0,366	8	8							unpaired t-test	t(14) = 2.539, **p = 0.0236**
2A	% MOMA+ cells vessel	12,2 ± 1,553	8	3	21,79 ± 1,865	7	3							unpaired t-test	t(13) = 3.986, **p = 0.0016**
2B	CD45hi Ly6C+ SiglecF-	12775 ± 3182	8	8	29179 ± 5429	10	10							unpaired t-test	t(16) = 2.438, **p = 0.0268**
2C	CD45hi Ly6Chi SiglecF-	4916 ± 1050	8	8	11207 ± 1763	10	10							unpaired t-test	t(16) = 2.872, **p = 0.0111**
2D	F4/80+ CD80+	4410 ± 375	8	8	7158 ± 751	10	10							unpaired t-test	t(15) = 3.024, **p = 0.0081**
2E	F4/80+ SiglecF- CD80+	3146 ± 167	8	8	6102 ± 743	10	10							unpaired t-test	t(16) = 3.485, **p = 0.0031**
2F	F4/80+ SiglecF+ CD80+	1262 ± 335	8	8	1049 ± 149	10	10							unpaired t-test	t(16) = 0.6229, p = 0.5421
3A	TNFa (Protein)	0,998 ± 0,105	7	7	1,869 ± 0,314	7	7							unpaired t-test	t(12) = 2.633, **p = 0.0218**
3B	CFTR (Protein)	1,000 ± 0,070	7	7	0,707 ± 0,064	8	8							unpaired t-test	t(13) = 3.104, **p = 0.0084**
3C	%CFTR+ F4/80+ SiglecF-	94,480 ± 0,453	5	5	85,180 ± 1,129	8	8							unpaired t-test	t(11) = 6.23, **p < 0.0001**
3D	%CFTR+ F4/80+ SiglecF+	81,420 ± 1,566	5	5	79,790 ± 1,846	8	8							unpaired t-test	t(11) = 0.6131, p = 0.5523
4A	%CFTR+ cells	42,030 ± 1,714	8	8	34,250 ± 2,110	8	8	40,87 ± 1,677	10	10				ANOVA + Dunnett´s	ANOVA F(2,23) = 4.961, **p = 0.0162**, HF vs. Sham **p = 0.0155**; HF vs. Treatment i.p. **p = 0.0299**; Brown-Forsythe F(2,23) = 5.377*10^-5, p >0.9999
4B	CFTR (Protein)							0,79 ± 0,156	6	6	1,011 ± 0,1508	8	8	unpaired t-test	t(12) = 1.009, p = 0.3328
4C	MFI CFTR+ cells							21227,00 ± 457,500	6	6	26054 ± 662,1	8	8	unpaired t-test	t(12) = 5.577, **p = 0.0001**
5A	Vessel wall thickness	6,473 ± 0,3042	3	3	9,785 ± 0,355	4	4	6,906 ± 0,3395	5	5				ANOVA + Dunnett´s	ANOVA F(2,9) = 25.71, **p = 0.0002**, HF vs. Sham **p = 0.0003**; HF vs. Treatment i.p. **p = 0.0003**; Brown-Forsythe F(2,9) = 0.154, p = 0.8511
5B	SMA (Protein)	1,01 ± 0,1203	8	8	3,153 ± 0,428	8	8	1,353 ± 0,2826	10	10				ANOVA + Dunnett´s	ANOVA F(2,23) = 13.63, **p = 0.0001**, HF vs. Sham **p = 0.0002**; HF vs. Treatment i.p. **p = 0.0006**; Brown-Forsythe F(2,23) = 2.317, p = 0.1212
5C	Vessel wall thickness				9,785 ± 0,355	4	4	5,09 ± 0,2631	4	4	5,905 ± 0,1477	4	4	ANOVA + Dunnett´s	ANOVA F(2,9) = 86.86, **p <0.0001**, HF vs. Treatment i.p. **p <0.0001**; HF vs. Treatment o.t. **p <0.0001**, Brown-Forsythe F(2,9) = 1.494, p = 0.2752
5D	SMA (Protein)				3,153 ± 0,428	8	8	1,353 ± 0,1783	6	6	1,148 ± 0,1067	8	8	ANOVA + Dunnett´s	ANOVA F(2,19) = 15.12, **p = 0.0001**, HF vs. Treatment i.p. **p = 0.0009**; HF vs. Treatment o.t. **p = 0.0001**; Brown-Forsythe F(2,19) = 3.491, p = 0.0511
S4	CFTR (Protein)	1 ± 0,0698	7	7	0,7071 ± 0,064	8	8	0,9064 ± 0,097	9	9				ANOVA + Dunnett´s	ANOVA F(2,21) = 3.239, p = 0.0594, HF vs. Sham **p = 0.0414**; HF vs. Treatment i.p. p = 0.1551; Brown-Forsythe F(2,21) = 1.370, p = 0.2759

Figure	Parameter	Circulating (S6A)	n	N	Pulmonary (S6B)	n	N
S6A/B	T-cells	48,22 ± 6,293	5	5	61 ± 12,85	5	5
	B-cells	67,76 ± 4,823	5	5	73,5 ± 8,25	5	5
	Ly6C+	97,42 ± 0,3891	5	5	91,1 ± 2,85	5	5
	Ly6Chi	82,28 ± 2,498	5	5	82,7 ± 5,25	5	5
	Neutrophils	78,12 ± 5,015	5	5	84,7 ± 6,9	5	5
	F4/80+ cells				90,2 ± 2,65	5	5
	Siglec neg cells				94,2 ± 1,6	5	5
	Siglec+ cells				81,3 ± 6,6	5	5
	Endothelial cells				99,6 ± 0,15	5	5
	Epithelial cells				99,6 ± 0,25	5	5

Figure	Parameter	Control	n	N	HF	n	N	Treatment (i.p.)	n	N	Treatment (o.t.)	n	N	Comparison Test	Statistical Outcome
S7A	# F4/80+ cells	113901 ± 41551	8	8	105412 ± 44551	10	10	178978 ± 81262	6	6	235305 ± 77666	8	8	Kruskal-Wallis + Dunn´s	H(3) = 19.36, **p = 0.0002**, HF vs. sham p >0.9999; HF vs. Treatment i.p. **p = 0.0518**; HF vs. Treatment o.t. **p = 0.0004**
S7B	# F4/80+ SiglecF- cells	79936 ± 28928	8	8	77975 ± 34374	10	10	151022 ± 71715	6	6	197345 ± 107979	8	8	Kruskal-Wallis + Dunn´s	H(3) = 19.60, p = **0.0002**, HF vs. Sham p >0.9999; HF vs. Treatment i.p. **p = 0.0375**; HF vs. Treatment o.t. **p = 0.0005**
S7C	# F4/80+ SiglecF+ cells	25219 ± 5404	8	8	21328 ± 3661	10	10	24357 ± 3956	6	6	43407 ± 6316	8	8	ANOVA + Dunnett´s	ANOVA F(3,28) = 4.176, **p = 0.0146**, HF vs. Sham p = 0.8963; HF vs. Treatment i.p. p = 0.9576; HF vs. Treatment o.t. **p = 0.0073**; Brown-Forsythe F(3,28) = 0.3626, p = 0.7805
6A	% CD80+ F4/80+ cells	4,04 ± 1,09	8	8	5,81 ± 3,06	10	10	4,555 ± 2,21	6	6	5,035 ± 3,285	8	8	Kruskal-Wallis + Dunn´s	H(3) = 10.65, **p = 0.0138**, HF vs. Sham **p = 0.0041**; HF vs. Treatment i.p. p = 0.2380; HF vs. Treatment o.t. p = 0.6812
6B	% CD80+ F4/80+ SiglecF- cells	3,71 ± 1,002	8	8	6,42 ± 3,42	10	10	4,48 ± 2,26	6	6	4,39 ± 1,578	8	8	Kruskal-Wallis + Dunn´s	H(3) = 15.77, **p = 0.0013**, HF vs. Sham **p = 0.0003**; HF vs. Treatment i.p. p = 0.157; HF vs. Treatment o.t. **p = 0.0312**
6C	% CD80+ F4/80+ SiglecF+ cells	5,465 ± 2,448	8	8	4,875 ± 2,608	10	10	5,34 ± 1,69	6	6	7,365 ± 8,715	8	8	Kruskal-Wallis + Dunn´s	H(3) = 7.288, p = 0.0633, HF vs. sham p >0.9999; HF vs. Treatment i.p. p >0.9999; HF vs. Treatment o.t. p = 0.0565
6D	% CD206+ F4/80+ cells	1,6 ± 0,543	8	8	1,15 ± 0,428	10	10	2,56 ± 1,513	6	6	2,44 ± 0,508	8	8	Kruskal-Wallis + Dunn´s	H(3) = 23.75, **p <0.0001**, HF vs. Sham p = 0.5324; HF vs. Treatment i.p. **p = 0.0002**; HF vs. Treatment o.t. **p = 0.0002**
6E	% CD206+ F4/80+ SiglecF- cells	1,885 ± 0,915	8	8	1,405 ± 0,528	10	10	2,74 ± 1,315	6	6	2,7 ± 0,69	8	8	Kruskal-Wallis + Dunn´s	H(3) = 20.97, **p = 0.0001**, HF vs. sham p = 0.4297; HF vs. Treatment i.p. **p = 0.0003**; HF vs. Treatment o.t. **p = 0.0008**
6F	% CD206+ F4/80+ SiglecF+ cells	0,4625 ± 0,0967	8	8	0,7003 ± 0,193	10	10	1,592 ± 0,4024	6	6	1,608 ± 0,3125	8	8	Brown-Forsythe + Dunnett´s T3	Brown-Forsythe ANOVA F(3,15.71) = 4.871, **p = 0.0139**, HF vs. Sham p = 0.6253; HF vs. Treatment i.p. p = 0.2167; HF vs. Treatment o.t. p = 0.0822
S9	IL-10 (mRNA)				0,6659 ± 0,15	7	7	1,74 ± 0,408	9	9				unpaired t-test	t(14) = 2.218, **p = 0.0436**

Figure	Parameter	Control	n	N	PMA	n	N	Treatment (24h)	n	N	Treatment (96h)	n	N	Comparison Test	Statistical Outcome
S10	%CFTR+ cells	99,7 ± 12,1	11	3	71,25 ± 6,47	10	3	81,8 ± 9,1	11	3	91,6 ± 21,1	11	3	Kruskal-Wallis + Dunn´s	H(3) = 26.20, **p <0.0001**, ctrl vs. PMA **p <0.0001**; ctrl vs. Lum 24h **p = 0.0010**; ctrl vs. Lum 96h p = 0.2817; PMA vs. Lum 24h p >0.9999 ; PMA vs. Lum 96h p = 0.0304 ; Lum 24h vs. Lum 96h p = 0.4643

Table	Parameter	Control	n	N	HF	n	N	Treatment (i.p.)	n	N	Before treatment	n	N	Comparison Test	Statistical Outcome
ST5A	EF [%]	64,25 ± 1,84	8	8	43 ± 3,313	10	10	41,9 ± 3,202	10	10				ANOVA + Tukey´s	ANOVA F(2,25) = 16.2, **p <0.0001**, Sham vs. HF **p = 0.0001**; Sham vs. Treatment **p <0.0001**; HF vs. Treatment p = 0.9611; Brown-Forsythe F(2,25) = 1.943, p = 0.1643
	SV [µl]	16,13 ± 1,093	8	8	26,9 ± 2,073	10	10	25,2 ± 1,618	10	10				ANOVA + Tukey´s	ANOVA F(2,25) = 0.2626, p = 0.7711, Sham vs. HF p = 0.9481; Sham vs. Treatment p = 0.927; HF vs. Treatment p = 0.7518; Brown-Forsythe F(2,25) = 0.6023, p = 0.5553
	CO [µl/min]	15,14 ± 0,891	8	8	15,3 ± 1,192	10	10	14,87 ± 0,918	10	10				ANOVA + Tukey´s	ANOVA F(2,25) = 0.04746, p = 0.9537, Sham vs. HF p = 0.9935; Sham vs. Treatment p = 0.9825; HF vs. Treatment p = 0.95; Brown-Forsythe F(2,25) = 0.4002, p = 0.6744
	EDV [µl]	40,75 ± 2,094	8	8	63,1 ± 2,536	10	10	62,2 ± 3,756	10	10				ANOVA + Tukey´s	ANOVA F(2,25) = 16.38, **p <0.0001**, Sham vs. HF **p <0.0001**; Sham vs. Treatment **p = 0.0001**; HF vs. Treatment p >0.9737; Brown-Forsythe F(2,25) = 3.148, p = 0.0603
	ESV [µl]	14,750 ± 1,386	8	8	36,1 ± 2,584	10	10	36,9 ± 3,686	10	10				ANOVA + Tukey´s	ANOVA F(2,25) = 17.52, **p <0.0001**, Sham vs. HF **p <0.0001**; Sham vs. Treatment **p <0.0001**; HF vs. Treatment p = 0.9774; Brown-Forsythe F(2,25) = 3.096, p = 0.0629
ST5B	EF [%]							41,9 ± 3,202	10	10	43,4 ± 3,618	10	10	paired t-test	t(9) = 0.5658, p = 0.5853
	SV [µl]							25,2 ± 1,618	10	10	26,5 ± 1,905	10	10	paired t-test	t(9) = 0.5826, p = 0.5745
	CO [µl/min]							14,87 ± 0,918	10	10	16,18 ± 1,112	10	10	paired t-test	t(9) = 1.108, p = 0.2965
	EDV [µl]							62,2 ± 3,756	10	10	63,6 ± 5,429	10	10	paired t-test	t(9) = 0.3144, p = 0.7604
	ESV [µl]							36,9 ± 3,686	10	10	37,2 ± 4,923	10	10	paired t-test	t(9) = 0.09036, p = 0.93

Table	Parameter	Control			n	N	HF			n	N	Treatment (i.p.)			n	N	Treatment (o.t.)			n	N	Comparison Test	Statistical Outcome
ST6a	All (CD80+ F4/80+)	4181	±	1798	8	8	6628	±	3153	10	10	9427	±	4609	6	6	13188	±	7203	8	8	Kruskal-Wallis + Dunn´s	H(3) = 9.691, **p = 0.0214**, sham vs. HF **p = 0.0117**; sham vs. Treatment i.p. p >0.9999; sham vs. Treatment o.t. p = 0.7583; HF vs. Treatment i.p. p = 0.636; HF vs. Treatment o.t. p = 0.9392; Treatment i.p. vs. Treatment o.t. p >0.9999
ST6b	Non-alveolar (CD80+ SiglecF- F4/80+)	3146	±	167	8	8	6102	±	743	10	10	7870	±	1242	6	6	9284	±	905	8	8	Brown-Forsynthe + Dunnett´s T3	Brown-Forsythe F(3,16.42) = 10.05, **p = 0.0005**, sham vs. HF **p = 0.0166**; sham vs. Treatment i.p. p = 0.0584; sham vs. Treatment o.t. **p = 0.0015**; HF vs. Treatment i.p. p = 0.7714; HF vs. Treatment o.t. p = 0.0887; Treatment i.p. vs. Treatment o.t. = 0.917
ST6c	Alveolar (CD80+ SiglecF+ F4/80+)	888,5	±	1396	8	8	1114	±	801	10	10	1297	±	826	6	6	2456	±	5084	8	8	Kruskal-Wallis + Dunn´s	H(3) = 14.78, **p = 0.002**, sham vs. HF p >0.9999; sham vs. Treatment i.p. p >0.9999; sham vs. Treatment o.t. **p = 0.01**; HF vs. Treatment i.p. p >0.9999; HF vs. Treatment o.t. p = 0.0028; Treatment i.p. vs. Treatment o.t. p = 0.0875
ST6d	All (CD206+ F4/80+)	1558	±	689	8	8	1076	±	479	10	10	4776	±	4806	6	6	5693	±	1319	8	8	Kruskal-Wallis + Dunn´s	H(3) = 24.84, **p <0.0001**, sham vs. HF p >0.9999; sham vs. Treatment i.p. p = 0.1616; sham vs. Treatment o.t. **p = 0.0156**; HF vs. Treatment i.p. p = 0.0031; HF vs. Treatment o.t. p <0.0001; Treatment i.p. vs. Treatment o.t. p >0.9999
ST6e	Non-alveolar (CD206+ SiglecF- F4/80+)	1534	±	734	8	8	1033	±	526	10	10	4346	±	2891	6	6	5074	±	676	8	8	Kruskal-Wallis + Dunn´s	H(3) = 24.78, **p <0.0001**, sham vs. HF p >0.9999; sham vs. Treatment i.p. p = 0.1651; sham vs. Treatment o.t. **p = 0.0131**; HF vs. Treatment i.p. p = 0.0038; HF vs. Treatment o.t. p <0.0001; Treatment i.p. vs. Treatment o.t. p >0.9999
ST6f	Alveolar (CD206+ SiglecF+ F4/80+)	93	±	27	8	8	121	±	34	10	10	340	±	65	6	6	718	±	193	8	8	Brown-Forsynthe + Dunnett´s T3	Brown-Forsythe F(3,9.096) = 8.033, **p = 0.0063**, sham vs. HF p = 0.9838; sham vs. Treatment i.p. **p = 0.0498**; sham vs. Treatment o.t. p = 0.073; HF vs. Treatment i.p. p = 0.0864; HF vs. Treatment o.t. p = 0.0902; Treatment i.p. vs. Treatment o.t. = 0.4029

Legend
Mean ± SEM
Mean ± SEM
Median ± IQR

Supplemental Table 5: A) Endpoint heart function parameters of sham, HF, and Lumacaftor treated HF mice and **B)** longitudinally assessed heart function parameters pre and post Lum treatment in HF mice. Values presented as mean ± SEM; In **A**, * denotes p ≤ 0.05 after one-way ANOVA with Tukey's *post hoc* testing; in **B**, single, paired comparisons using t-test with exact P-value computation. *CO – cardiac output, EDV – end diastolic volume, EF – ejection fraction, ESV – end systolic volume, HF – heart failure, Lum – Lumacaftor, LVM – left ventricular mass, SV – stroke volume.*

A

	**Sham**	**HF + vehicle**	**HF + Lum i.p.**
EF [%]	64.8 ± 1.7	39.6 ± 2.5 *	39.3 ± 3.2 *
SV [µl]	26.6 ± 1.1	25.1 ± 2.1	24.4 ± 1.9
CO [ml/min]	15.5 ± 0.9	14.7 ± 1.1	14.4 ± 1.1
EDV[µl]	41.1 ± 1.9	63.6 ± 3.1 *	63.8 ± 4.4 *
ESV [µl]	14.6 ± 1.2	38.3 ± 1.9 *	39.3 ± 4.0 *

B

	**HF pre Lum (i.p.)**	**HF post Lum (i.p.)**	**P-value**
EF [%]	41.9 ± 3.2	43.4 ± 3.6	0.5853
SV [µl]	25.2 ± 1.6	26.5 ± 1.9	0.5745
CO [ml/min]	14.9 ± 0.9	16.2 ± 1.1	0.2965
EDV[µl]	62.2 ± 3.8	63.6 ± 5.4	0.7604
ESV [µl]	36.9 ± 3.7	37.2 ± 4.9	0.9300

Supplemental Table 6: Pulmonary macrophage cell numbers of sham, HF, and Lumacaftor treated HF mice. Numbers per 10^6 viable $CD45^+$ cells. In **a, c, d, e,** data expressed as median ± IQR; *denotes p ≤ 0.05 significant difference to sham, $ denotes significant difference to HF mice after Kruskal Wallis with Dunn's *post-hoc* testing. In **b, f**, data expressed as mean ± SEM; *denotes p ≤ 0.05 significant difference to sham mice after Brown-Forsythe ANOVA with Dunnett's *post-hoc* testing. *CD – cluster of differentiation, HF – heart failure, i.p. – intraperitoneal, o.t. – orotracheal*

	**Macrophage type**	**Sham**	**HF**	**i.p.**	**o.t.**
**a**	**All ($CD80^+$ $F4/80^+$)**	4181 ± 1798	6628 ± 3153*	9427 ± 4609	13188 ± 7203
**b**	**non-alveolar ($CD80^+$ $SiglecF^-$ $F4/80^+$)**	3146 ± 167	6102 ± 743 *	7870 ± 1242	9284 ± 905 *
**c**	**alveolar ($CD80^+$ $SiglecF^+$ $F4/80^+$)**	889 ± 1396	1114 ± 801	1297 ± 826	2456 ± 5084 *$
**d**	**All ($CD206^+$ $F4/80^+$)**	1558 ± 689	1076 ± 479	4776 ± 4806 $	5693 ± 1319 *$
**e**	**non-alveolar ($CD206^+$ $SiglecF^-$ $F4/80^+$)**	1534 ± 734	1033 ± 526	4346 ± 2891 $	5074 ± 676 *$
**f**	**alveolar ($CD206^+$ $SiglecF^+$ $F4/80^+$)**	93 ± 27	121 ± 34	340 ± 65 *	718 ± 193

SUPPLEMENTAL FIGURES

Supplemental Figure 1 - Schematic overview of the experimental setup. *CFTR – cystic fibrosis transmembrane regulator*.

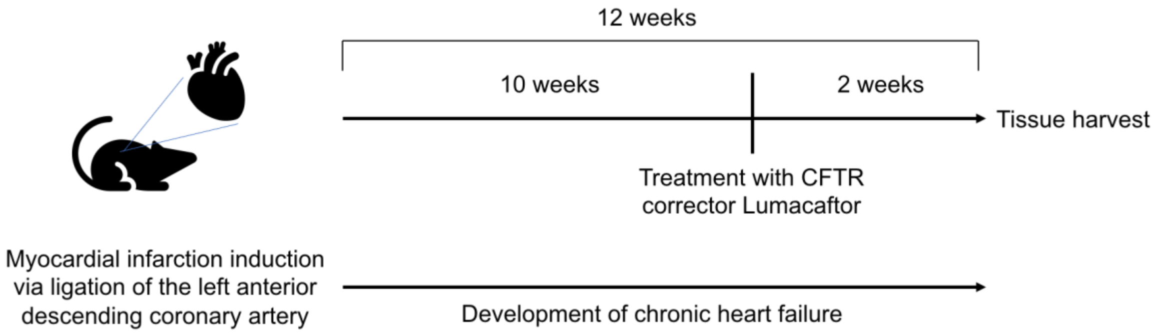

Supplemental Figure 2 - Gating strategy for pulmonary macrophages. *CD – cluster of differentiation, FSC – forward scatter, SSC – sideward scatter*

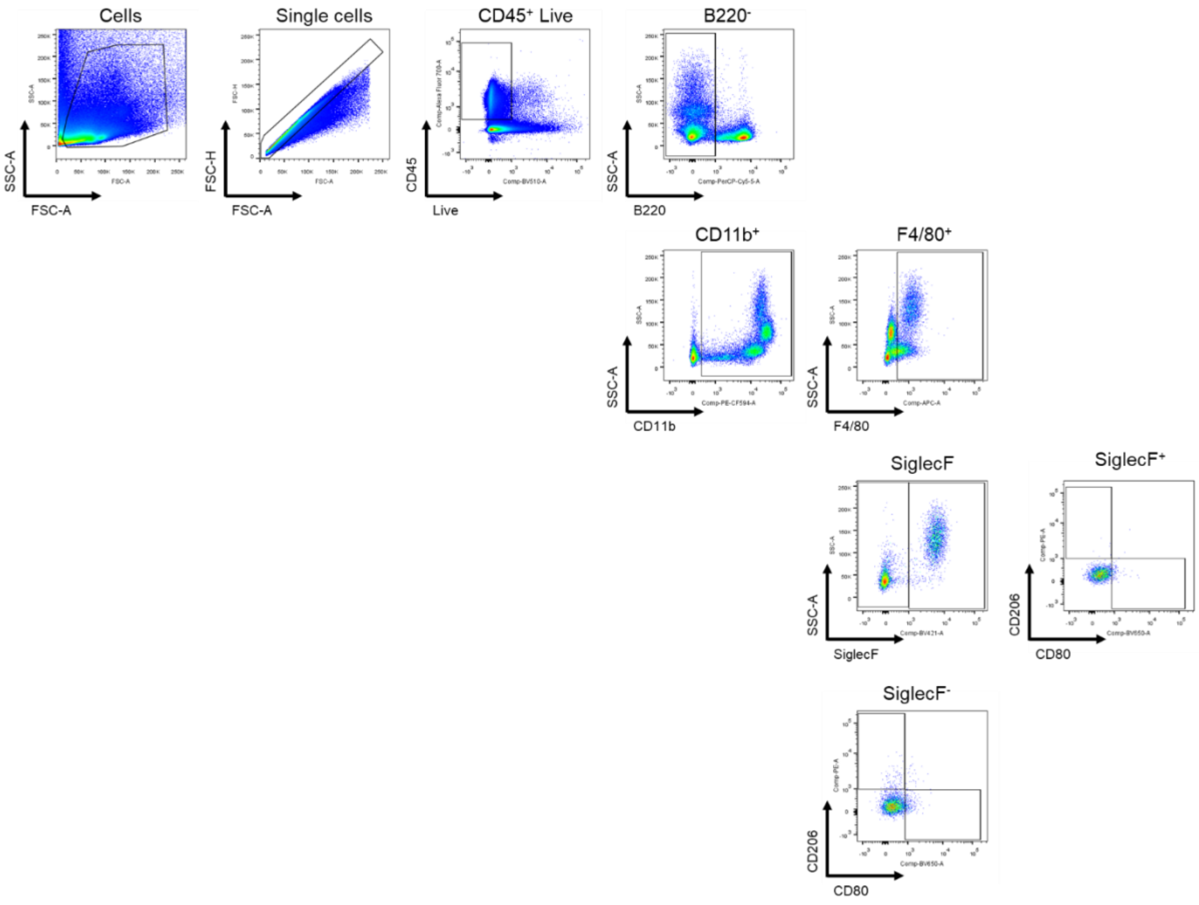

Supplemental Figure 3 - Heart failure associates with vascular remodelling in the lung. Higher smooth muscle actin (*Acta2*) gene expression in the HF lung compared to sham. N=8; data expressed as mean ± SEM. * denotes p ≤ 0.05 after unpaired t-test. *HF – heart failure.*

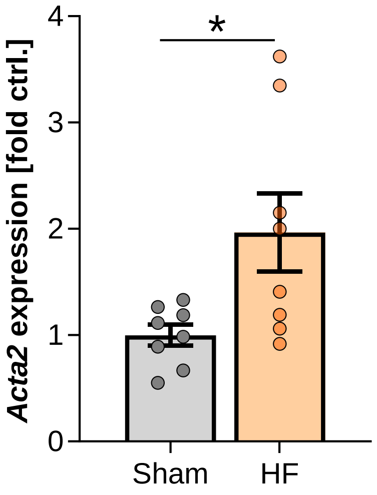

Supplemental Figure 4: Representative western blot and quantification of the CFTR expression in the lungs of sham, heart failure (HF), and Lumacaftor (Lum)-treated HF mice. Data expressed as mean ± SEM; * denotes p ≤ 0.05 relative to HF one-way ANOVA with Dunnett's *post-hoc* testing. *HF – heart failure, Lum – lumacaftor.*

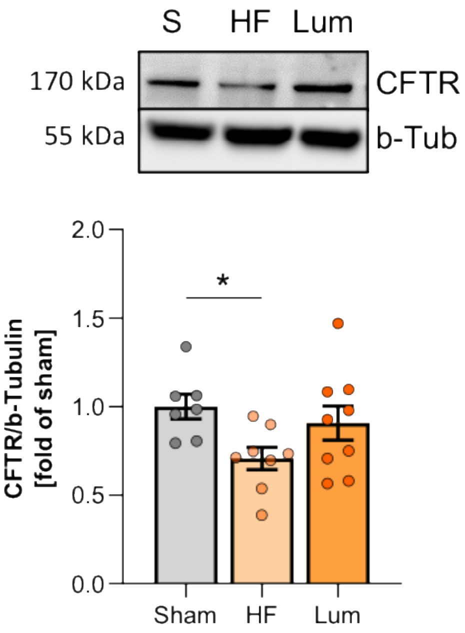

Supplemental Figure 5: Representative images of the vessel wall thickness in the lungs of sham, HF and Lum-treated (i.p. or o.t.) HF mice, Haematoxylin and Eosin staining, arrows indicating vessel walls, scale bar 20 µm. *HF – heart failure, i.p. – intraperitoneal, Lum - lumacaftor, o.t. – orotracheal*

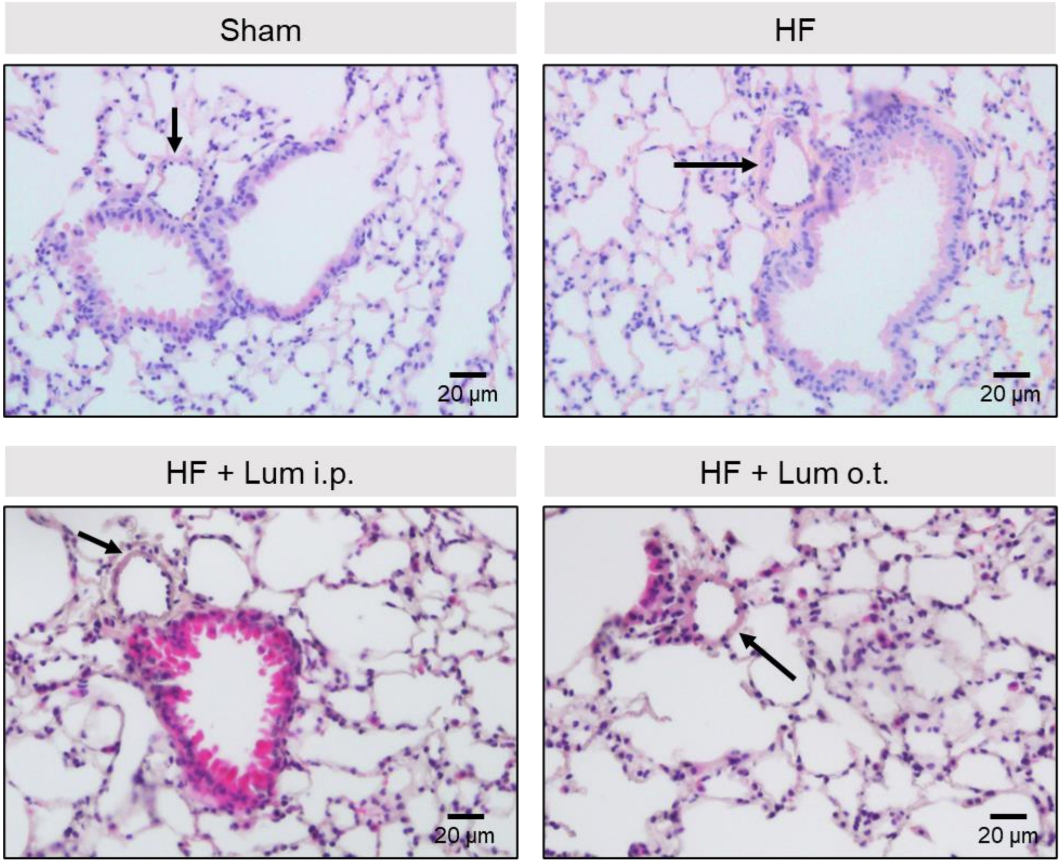

Supplemental Figure 6 - Cell type-specific cystic fibrosis transmembrane regulator expression. Flow cytometric assessment of CFTR surface expression on **A)** circulating immune cells subsets and **B)** immune and non-immune cells in the lung of naïve mice. N=5; for **(A)**, data expressed as mean ± SEM; for **(B)**, data is expressed as median ± IQR. N denotes number of independent biological replicates. *CFTR - cystic fibrosis transmembrane regulator, hi – high, Ly6C – lymphocyte antigen 6C, neg – negative*

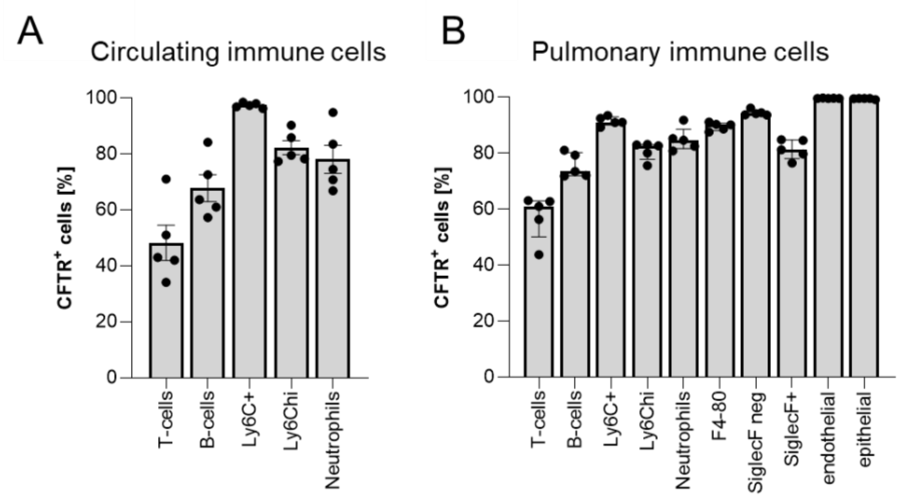

Supplemental Figure 7 - Cystic fibrosis transmembrane regulator correction increases the number of pulmonary macrophages. A) Number of F4/80$^+$ pulmonary macrophages of sham, HF, and Lum-treated (i.p. or o.t.) HF mice. **B)** Number of SiglecF$^-$ pulmonary F4/80$^+$-macrophages of sham, HF, and Lumacaftor treated (i.p. and o.t.) HF mice. **C)** Number of SiglecF$^+$ pulmonary F4/80$^+$-macrophages of sham, HF, and Lumacaftor treated (i.p. and o.t.) HF mice. N denotes number of independent biological samples (N =8 for Sham, N=10 for HF, N=6 for HF + Lum i.p., N=8 for HF + Lum o.t.). **D)** Representative dot plots of SiglecF and F4/80 expression of CD11b$^+$ pulmonary lymphocytes of sham, HF, and Lum-treated (i.p. and o.t.) HF mice. In **A, B**, data expressed as median ± IQR; *denotes $p \leq 0.05$ relative to HF after Kruskal Wallis with Dunn's *post-hoc* testing. In **C**, data expressed as mean ± SEM; *denotes $p \leq 0.05$ relative to HF after one-way ANOVA with Dunnett's *post-hoc* testing. *CD – cluster of differentiation, HF – heart failure, i.p. – intraperitoneal, Lum - lumacaftor, o.t. – orotracheal*

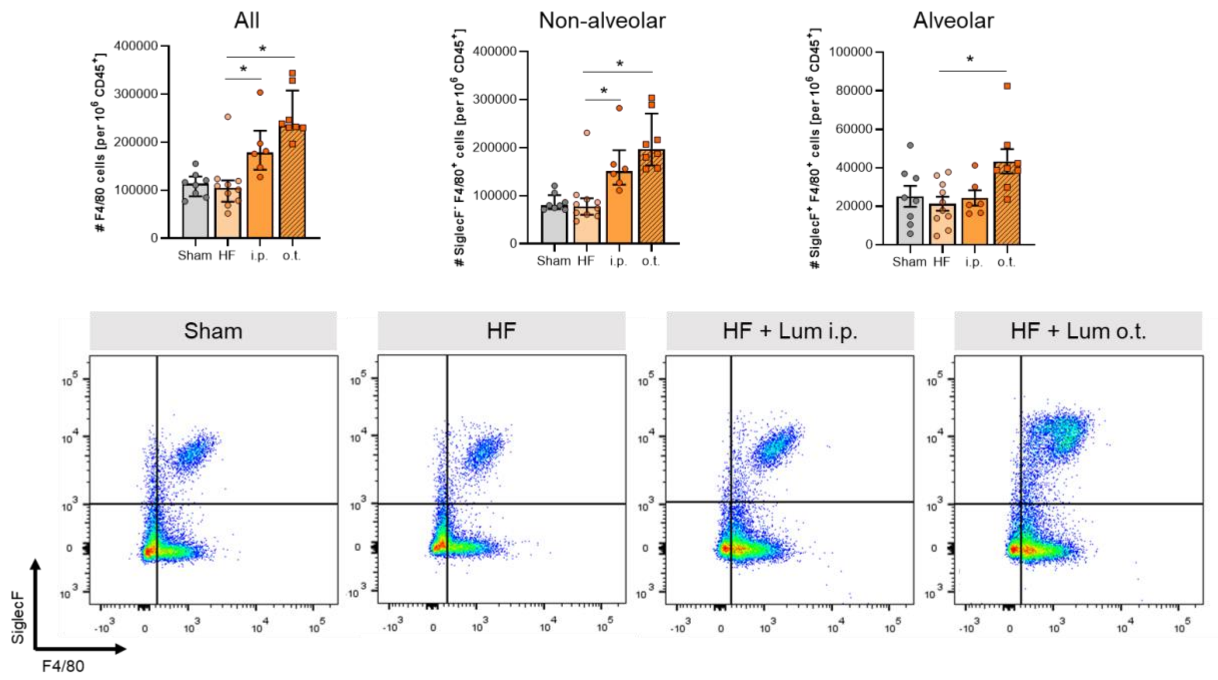

Supplemental Figure 8 - Representative dot plots of the CD80 and CD206 expression of **A)** SiglecF$^-$ and **B)** SiglecF$^+$ pulmonary F4/80$^+$ macrophages of sham, HF and Lum-treated (i.p. or o.t.) HF mice. CD - cluster of differentiation, *HF – heart failure, i.p. – intraperitoneal, Lum - lumacaftor, o.t. – orotracheal*

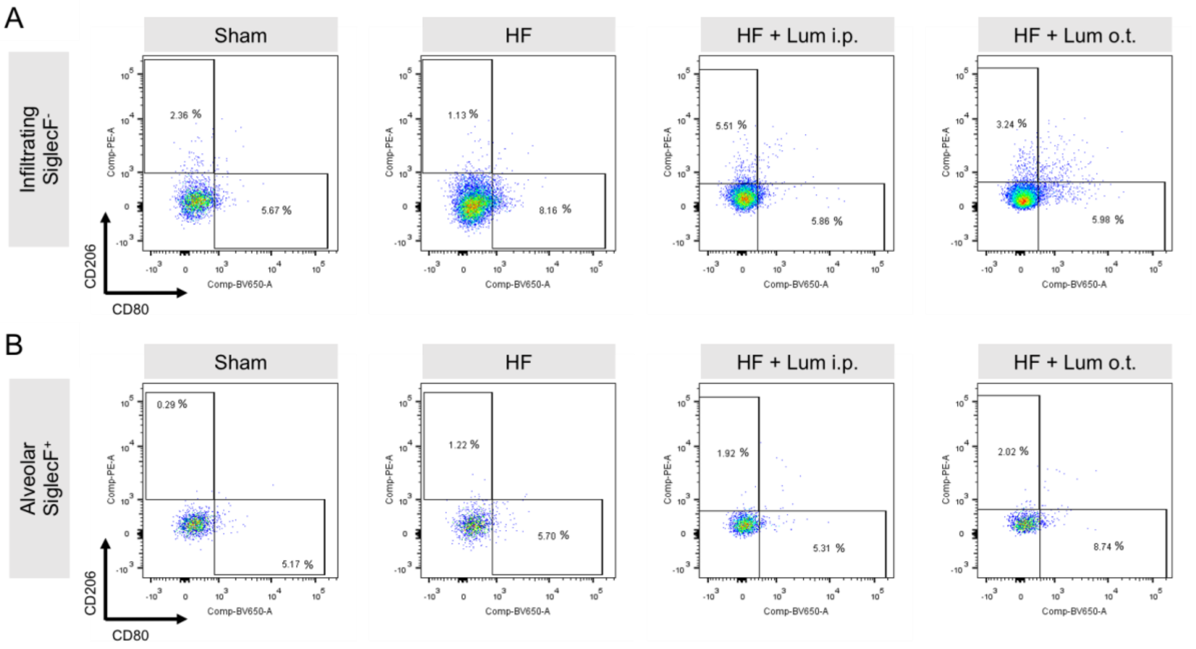

Supplemental Figure 9 - Cystic fibrosis transmembrane regulator correction augments anti-inflammatory cytokine levels in the lung of heart failure mice. Treatment with Lum during HF leads to an increase in IL-10 gene expression in the lung. N denotes number of independent biological samples (N=7 for HF, N=9 for HF + Lum i.p.); data expressed as mean ± SEM. * denotes $p \leq 0.05$ after unpaired t-test. *Ctrl – control, HF – heart failure, IL – interleukin, Lum - lumacaftor*

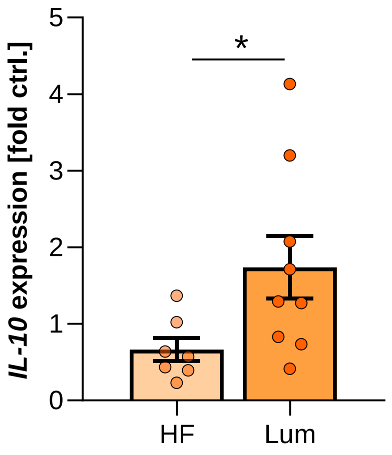

Supplemental Figure 10: Activation of macrophages reduces their cystic fibrosis transmembrane regulator surface expression. PMA-induced CFTR down-regulation in mouse macrophages (RAW264.7 cells) is attenuated by treatment with CFTR corrector Lum assessed by flow cytometry. N=3 with n=3-4 biological replicates; data expressed as median ± IQR; * denotes significant difference to control and $ denotes significant difference to PMA after Kruskal Wallis followed by Dunn's *post-hoc* testing. CFTR – cystic fibrosis transmembrane regulator, *Ctrl – control, Lum – lumacaftor, PMA* - Phorbol 12-myristate 13-acetate.

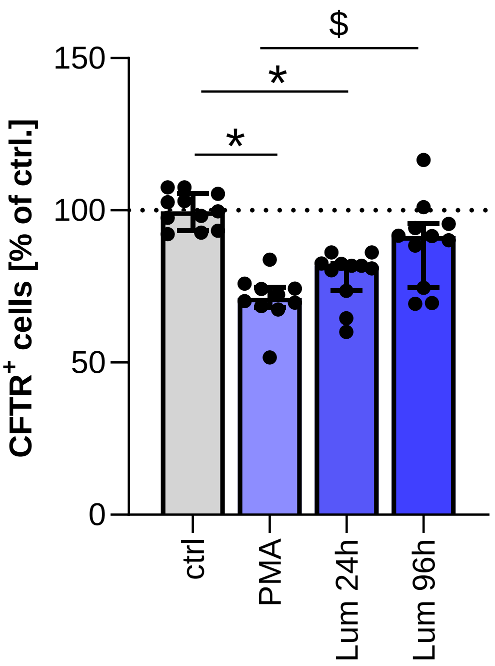

Supplement: Supplementary file 1 [file DataSheet_1.pdf]
